# A Systematic Review of Zoonotic Enteric Parasites Carried by Flies, Cockroaches, and Dung Beetles

**DOI:** 10.3390/pathogens11010090

**Published:** 2022-01-13

**Authors:** Avi Patel, Meg Jenkins, Kelly Rhoden, Amber N. Barnes

**Affiliations:** 1 Stanton College Preparatory School, Jacksonville, FL 32209, USA; amber.n.barnes@gmail.com; 2 Department of Public Health, Brooks College of Health, University of North Florida, Jacksonville, FL 32224, USA; jenkins.meg@gmail.com (M.J.); k.rhoden@unf.edu (K.R.)

**Keywords:** zoonoses, parasites, one health, water, sanitation, hygiene

## Abstract

Filth flies, cockroaches, and dung beetles have been close neighbors with humans and animals throughout our joint histories. However, these insects can also serve as vectors for many zoonotic enteric parasites (ZEPs). Zoonoses by ZEPs remain a paramount public health threat due to our close contact with animals, combined with poor water, sanitation, and hygiene access, services, and behaviors in many global regions. Our objective in this systematic review was to determine which ZEPs have been documented in these vectors, to identify risk factors associated with their transmission, and to provide effectual One Health recommendations for curbing their spread. Using PRISMA guidelines, a total of 85 articles published from 1926 to 2021 were reviewed and included in this study. Qualitative analysis revealed that the most common parasites associated with these insects included, but were not limited to: *Ascaris* spp., *Trichuris* spp., *Entamoeba* spp., and *Cryptosporidium* spp. Additionally, prominent risk factors discovered in the review, such as poor household and community WASH services, unsafe food handling, and exposure to domestic animals and wildlife, significantly increase parasitic transmission and zoonoses. The risk of insect vector transmission in our shared environments makes it critically important to implement a One Health approach in reducing ZEP transmission.

## 1. Introduction

Flies (Diptera), cockroaches (Blattodea), and dung beetles (Coleoptera) share their environment with humans, animals, and other insects. While their presence can be beneficial—for example, through pollination, management of other pests, as a food source, and as an organic disposal system for decaying matter—they can also pose risks to human and animal health. Our close ecological connection to these insects presents the public health risk of disease transmission when one or more vectors are infected or contaminated with pathogenic organisms, such as zoonotic enteric parasites (ZEPs) [1,2,3,4,5]. ZEPs can be transmitted through direct contact with an insect vector harboring or carrying a parasite, or by accidental fecal–oral ingestion from contaminated food, water, hands, surfaces, and fomites [1].

Flies, particularly filth flies, are synanthropic and can be found anywhere humans are present, particularly in areas with poor water, sanitation, and hygiene services and practices [6,7]. Of the 46 species of flies that are associated with unclean environments or conditions of “filth”, 21 species are considered “disease-causing flies” or known to be vectors of foodborne pathogens [8] (p. 199). Many species of filth flies are coprophagic, feeding on the fecal waste of animals and humans. While these insects often favor indoor spaces, they frequently move back and forth between contaminated environmental settings and human living spaces. This repeated contact introduces the risk of exposure to enteric diseases of public health concern [1,8]. Filth flies are drawn to damp, organic matter (e.g., prepared food, garbage, sewage, or feces) to feed and lay eggs [7]. These behaviors create the risk of transmission of a variety of bacteria, viruses, and parasites that are shed in excreta [2,5,7,9]. As filth flies land on materials which can host a variety of pathogens and parasites, they are able to mechanically collect infectious particles or parasitic oocysts on their legs, bodies, or mouthparts as well as ingest the pathogen. Transmission to humans or animals occurs mechanically through contact with shared surfaces or other items, hands and faces, or food products [1,7,8].

While over 3500 species of cockroaches exist worldwide, only thirty species are known to be associated with human habitation [10]. Cockroaches have strong nocturnal habits and are often prevalent in areas of significant warmth, moisture, darkness, and where they can access exposed food particles [3]. Cockroaches commonly exist in the residential domain, but can also be found in restaurants, grocery stores, hospitals, and commercial facilities [11]. In addition to the triggering of asthma and other respiratory conditions due to residential infestations, cockroaches have been found to harbor parasitic microorganisms, externally on their cuticle or internally in their gastrointestinal tract [12]. They have been known to spread multiple pathogens, including bacteria, protozoa, fungi, and pathogenic intestinal worms [1,10]. Human consumption of vector-contaminated food is a risk factor for foodborne illness and the acquisition of parasitic infections. Additionally, human consumption of cockroaches, directly as entomophagy or accidentally, can also represent a risk of infection that is of public health importance [13]. Due to cockroaches’ feeding habits and preferences for human food and feces, they have the potential to become mechanical vectors for the spread of various zoonotic enteric parasites [3].

Dung beetles are coprophagous insects that depend on the fecal material of vertebrates in order to consume and reproduce. Dung beetles are found worldwide, including in places such as farms, peri-urban regions, and urban areas [14]. More than 7000 species of dung beetles handle, bury, or move wildlife feces every day [15]. Dung beetles may transmit disease mechanically on their exoskeletons or within their gastrointestinal systems [1,15]. Through contact with feces from a variety of sources such as livestock and companion animals as well as wild animals, dung beetles pose the threat of ZEP transmission of these parasites to humans. Dung beetles may also further spread disease in urban areas where unmanaged fecal waste is prevalent, such as communities with poor sanitation measures and areas where livestock and domestic animals live near humans [14]. 

The aim of this study was to determine which zoonotic enteric parasites (ZEPs) have been reported in filth flies, cockroaches, and dung beetles, and to identify the risk factors associated with their transmission. Understanding more about these vectors of public health importance will inform opportunities for One Health research, guidance, intervention, and collaboration.

## 2. Methods

### 2.1. Search Strategy

Between 28 January and 4 February 2021, we initially searched the following databases: Pubmed; Web of Science Core Collection; Google Scholar; Environment Complete; Science Direct; GALE databases of Agriculture Collection; Nursing and Allied Health Outcomes; Environmental Studies and Policy; the ProQuest databases of the ABI/INFORM Collection; Agricola, Earth, Atmospheric, and Aquatic Sciences Collection; Agricultural and Environmental Science Collection; Health and Medicine; MEDLINE (Proquest); and TOXLINE. Search strings were developed for each database using keywords related to filth flies, cockroaches, and dung beetles in conjunction with zoonotic enteric parasites [16]. When the option was available, database search results were further restricted to journal articles and the title and abstract keywords only. Accessible results were copied into the citation manager Refworks by database, and a master folder was created for all titles found in the initial search. An updated search was conducted on 27 December 2021 for any relevant titles published in the months following the initial search using the same search parameters as above. An informal protocol with the full listing of all search strings used and their corresponding database is provided in Supplementary Materials Table S1. A formal protocol was not prepared and the review was not registered.

### 2.2. Screening Process and Study Selection

Following PRISMA guidelines, titles were screened first for eligibility based on full and legible citations and journal article titles only [17]. Then, in groups of two reviewers at a time, the titles and abstracts were assessed. Inclusion criteria consisted of titles that were: (a) peer-reviewed journal articles; (b) from any publication year; with (c) primary research documenting the presence of a recognized or probable ZEP in an insect vector, either through natural or experimental infection; and the (d) ZEP has a primarily enteric transmission route. Exclusion criteria comprised: (a) any publication that was not a peer-reviewed journal article; (b) titles written in a language other than English without relevant information provided in an English abstract; (c) reviews or models that did not contain primary research; (d) research on vectors other than filth flies, cockroaches, or dung beetles; (e) research on enteric or gastrointestinal parasites that are not considered zoonotic or likely to be zoonotic; or (f) research that included negative results. When an abstract was not available for the first round of screening, the title was included in the next round for full text review. Titles that studied zoonotic enteric parasites not on our initial list of search terms were included after review by the team against the criteria outlined above.

Full text documents were retrieved by the authors and through the assistance of university librarians. Each full text title was reviewed by at least two authors based on the eligibility criteria above and subsequently marked for inclusion or exclusion. The senior author (AB) served as a tie-breaker when needed. If more than one title addressed the same study or data, the more complete publication was retained for inclusion. When the full text of the article was written in a language other than English, the titles were retained if the relevant inclusion criteria were met in an available English abstract. A second round of review was performed on the excluded full-text articles as a quality control measure in order to ensure a comprehensive list of final studies for inclusion.

### 2.3. Data Extraction

A qualitative analysis was conducted on the included studies by the reviewers to account for the wide variety of publication styles and research methods presented. From the included studies, data were extracted to determine the publication year, the location of the study site, the source or location of the samples, the vector(s) analyzed, the parasite(s) analyzed, specific prevalence rates, if provided, and the means through which the vector was infected (natural or experimental). Information on risk factors for human or animal transmission outlined in the article was also noted.

## 3. Results

**Figure 1:**
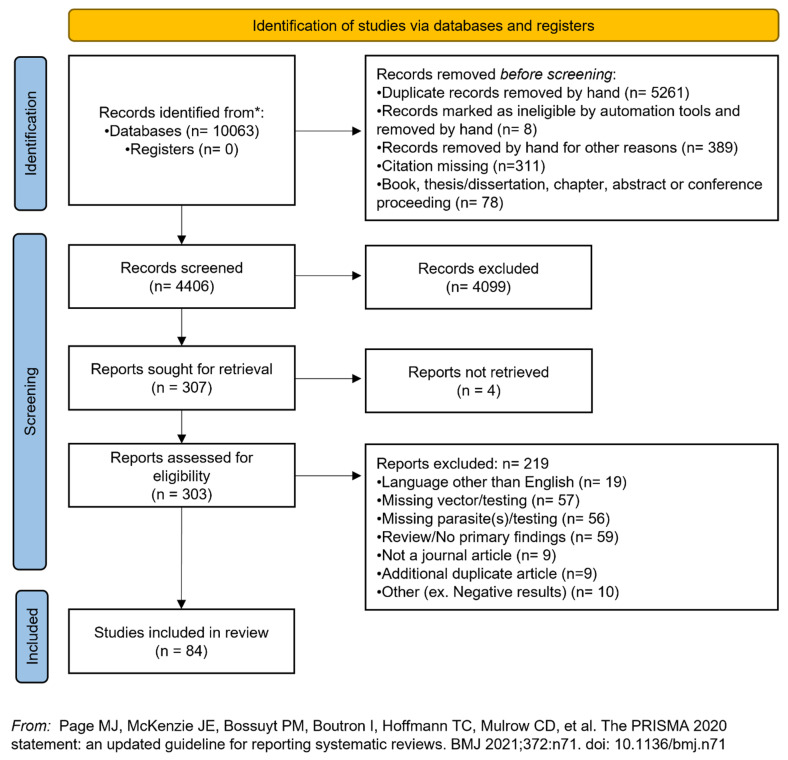
PRISMA screening flowchart of study selection.

The full search resulted in 10,063 accessible titles. We removed 5261 duplicate records, 8 titles that were not legible with the use of automation tools, 311 records with missing citations, and 78 books, book chapters, abstracts, thesis/dissertations, and conference proceedings. At this point, 4406 records remained for the title and abstract screening, and 4099 were excluded using the eligibility criteria outlined above. We attempted to find the full-text versions of 307 titles, but 4 records were not accessible through institutional library channels. A total of 303 articles were read in their entirety, if written in English, or the abstract was reviewed if the full text was not available in English. All titles at this stage were screened against the inclusion/exclusion criteria, and 85 titles were incorporated into the final tally of the study results. Full-text articles were excluded for language (n = 19, missing vector or vector not tested (n = 57), missing zoonotic enteric parasite or not testing for parasite (n = 55), article was a review or did not have primary findings (n = 59), the publication was not a journal article (n = 9), the title was an additional duplicate not removed at the earlier stage (n = 9), or other reasons such as negative results (n = 10). A PRISMA flow diagram of the screening process is available in Figure 1.

Studies were conducted worldwide, across countries on every continent, except for Antarctica (n = 85; Table 1). The most common included the United States (n = 13), Nigeria (n = 7), Ethiopia (n = 5), and Poland (n = 5). More broadly, studies were conducted in the continental regions of North America (n = 13), South America (n = 13), Europe (n = 20), Africa (n = 17), Asia (n = 19), and Australia (n = 2). Publication dates ranged from 1926 through 2021. Several of the studies (n = 11) identified met the inclusion criteria based on an English abstract and were conservatively included in the results. However, the full text could not be analyzed due to language limitations of the authors. 

### Zoonotic Enteric Parasites and Vectors Included in Review

**Figure 2:**
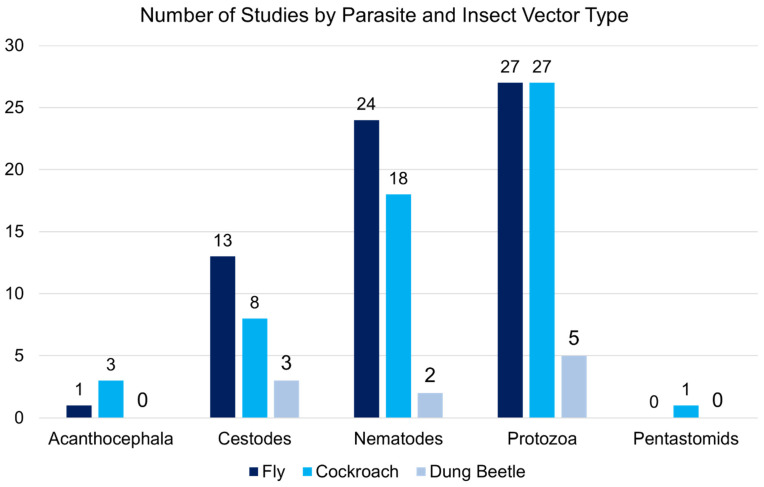
Number of included studies conducted on each insect vector by parasite category.

Most research was conducted on flies (n = 46), followed by cockroaches (n = 33) and dung beetles (n = 8). Two studies investigated two vectors at the same time (cockroaches and flies). The types of infection found in the insect vectors included natural (n = 49), experimental (n = 26), and mixed (n = 10). Parasites varied by different types and/or species classifications of the larval forms of protozoa (n = 8), the metacestodes stage (larva) of cestodes (n = 5), juvenile or larval nematodes (n = 14), acanthocephalans (n = 1), and pentastomids (n = 1). Protozoal species included *Entamoeba histolytica* (Schaudinn, 1903), *Entamoeba dispar* (Brumpt, 1925), *Entamoeba moshkovskii* (Tshalaia, 1941), *Balantidium coli* (Malmsten, 1857), *Cryptosporidium parvum* (Tyzer, 1912), *Giardia lamblia* (Kofoid and Christiansen, 1915) and *Giardia intestinalis* (Lambl, 1859), *Toxoplasma gondii* (Nicolle and Manceaux, 1908), *Sarcocystis muris* (Miescher, 1843), *Cyclospora cayetanensis* (Ortega, Gilman and Sterling, 1994), and *Blastocystis hominis* (Alexieff, 1911). Cestode species mentioned in the studies were *Echinococcus granulosus* (Batsch, 1786), *Taenia saginata* (Goeze, 1782), *Taenia hydatigena* (Pallas, 1766), *Taenia solium* (Linnaeus, 1758), *Dipylidium caninum* (Linnaeus, 1758), *Hymenolepis nana* (Bilharz, 1851), *Hymenolepis diminuta* (Rudolphi, 1819), and *Mesocestoides lineatus* (Goeze, 1782). Many species of nematodes were described in the included studies such as *Ancylostoma duodenale* (Dubini, 1843), *Necator americanus* (Stiles, 1902), *Trichuris suis* (Schrank, 1788), *Trichuris vulpis* (Froelich, 1789)*, Trichuris trichiura* (Linnaeus, 1771), *Ascaris lumbricoides* (Linnaeus, 1758)*, Ascaris suum* (Goeze, 1782), *Baylisascaris procynois* (Stefanski and Zarnowski, 1951), *Toxascaris leonine* (von Linstow, 1902), *Toxocara canis* (Werner, 1782), *Toxocara cati* (Schrank, 1788), *Trichinella spiralis* (Owen, 1835), *Physaloptera turgida* (Rudolphi, 1819), *Capillaria hepatica* (Bancroft, 1893), *Strongyloides stercoralis* (Bavay, 1876), *Strongyloides ransomi* (Schwartz and Alicata, 1930), *Setaria equina* (Abildgaard, 1789), *Syphacia obvelata* (Rudolphi, 1802), *Enterobius vermicularis* (Linnaeus, 1758), and *Oesophagostomus dentatum* (Rudolphi, 1803). An acanthocephalan species, *Moniliformis* (Bremser, 1811) was also named in the study results. The number of studies also varied by insect vector and parasite category, with most of the work investigating protozoa and nematodes in flies and cockroaches (Figure 2).

Many fly species were examined for zoonotic enteric parasites, particularly flies of public health importance from the Muscidae, Sarcophagidae, and Calliphoridae families. The vector fly species most often identified in the included titles was *Musca domestica* (Linnaeus, 1758). Additional fly species commonly studied among the included titles were *Chrysomya megacephala* (Fabricius, 1794), *Musca sorbens* (Wiedemann, 1830), *Stomoxys calcitrans* (Bishop, 1913), *Lucilia cuprina* (Meigen, 1826), and *Calliphora vicina* (Robineau-Desvoidy, 1830). 

Studies detailing the most common species of pathogens found on or in filth flies included: *Ascaris* spp. (n = 19), *Trichuris* spp. (n = 16), *Giardia* spp. (n = 13), *Cryptosporidium* spp. (n = 13), intestinal or non-specific hookworm (n = 10), *Taenia* spp. (n = 8), *Hymenolepis* spp. (n = 7), *Entamoeba* spp. (n = 7), *Toxocara* spp. (n = 9), and *T. gondii* (n = 3). The specimen locations of the fly samples were largely farms and/or pastures and fields (n = 16), waste disposal areas (n = 15), open markets and other food markets (n = 8), slaughterhouses or animal butcher areas (n = 6), households (n = 6), and schools and/or universities (n = 4). However, many included titles had received or reared fly samples in laboratory settings (n = 14).

While several species of cockroach were investigated among the titles for the presence of zoonotic enteric parasites, the two most common species examined in the included titles in this study were the German cockroach (*Blattella germanica*; Linnaeus, 1767) and the American cockroach (*Periplaneta americana*; Linnaeus, 1758). However, additional species were also studied such as *Periplaneta brunnea* (Burmeister, 1838), the Cuban burrowing cockroach (*Byrsotria fumigata*; Guérin-Méneville, 1857), the Madagascar hissing cockroach (*Gromphadorhina portentosa*; Schaum, 1853), the North American wood roach (*Paracoblatta* spp.), the oriental cockroach (*Blatta orientalis*; Linnaeus, 1758), the Turkestan cockroach (*Shelfordella lateralis*; Walker, 1868), the Australian cockroach (*Periplaneta australasiae*; Fabricius, 1775), the speckled cockroach (*Nauphoeta cinerea*; Oliver, 1789), among others.

Within the studies, parasitic pathogens were examined in or on cockroaches. These studies documented the *Ascaris* spp. (n = 12), *Trichuris* spp. (n = 10), *Entamoeba* spp. (n = 13), *Cryptosporidium* spp. (n = 7), *Blastocystis* spp. (n = 7), *Taenia* spp. (n = 6; one study may have also been Echinococcus spp.), *Balantidium coli* (n = 6), *Toxocara* spp. (n = 6), *Strongyloides* spp. (n = 5; in one study listed as *Strongyloides*-like nematodes), intestinal or non-specific hookworm (n = 4), *Giardia* spp. (n = 4), *Hymenolepis* spp. (n = 4), and *T. gondii* (n = 4). Cockroach specimens largely came from households (n = 11), specifically kitchen areas of living spaces (n = 4), hospitals (n = 5), open markets and other food markets (n = 4), schools/universities (n = 4). Many titles used cockroach specimens reared in laboratories (n = 8).

Several species of the dung beetle were studied by the included titles to determine if they could harbor, and potentially spread, zoonotic enteric parasites. The dung beetles were from the Scarabaeidae and Geotrupidae families, which primarily feed on fecal or decaying matter. Species came from the *Onthophagus* genus (e.g., *O. fracticornis*; Preyssler, 1790), the *Bubas* genus (e.g., *B. bison*; Linnaeus, 1767), the *Aphodius* genus including *A. rufus* (Moll, 1782) and *A. fimetarius* (Linnaeus, 1758), and the *Anoplotrupes* genus (e.g., *A. stercorosus*; Scriba, 1791), among others.

Within the titles that examined dung beetles, several parasite pathogens were found to have positive results. These zoonotic enteric parasites found on dung beetles included: *Taenia* spp. (n = 4), *Cryptosporidium* spp. (n = 2), *Ascaris* spp. (1), intestinal hookworm (n = 1), *Trichuris* spp. (n = 1), *Gongylonema* spp. (n = 1), and *Rhabditis* spp. (n = 1). Dung beetle samples were largely collected from farms, pastures, and fields (n = 5). The studies investigated natural infection (n = 2), used experimental design (n = 5), or a mixed-method approach (n = 1).

Numerous risk factors were mentioned for human and/or animal infection or exposure to zoonotic enteric parasites through insect vectors (Table 2). These included poor or inadequate water and sanitation services at home or in the community space (n = 27), having an open defecation site (n = 12) or unmanaged animal waste (n = 16) nearby, insufficient environmental hygiene or the absence of services such as garbage removal (n = 26), seasonal or climatic conditions preferred by the insect vector (n = 14), improper and unsafe food hygiene and storage (n = 23), insect behaviors and feeding practices (n = 29), direct animal contact (n = 22), and ingestion of infected vectors (n = 9).

## 4. Discussion

This review highlights the risk of ZEP transmission from insect vectors of interest, including flies, cockroaches, and dung beetles. Flies and cockroaches represent a significant hazard of being exposed to parasites in households and community spaces due to their synanthropic nature [1]. Close cohabitation with humans, especially in the household setting, poses an increased risk of transmission of ZEPs that can be compounded by other factors such as poor sanitation and hygiene. Alternatively, while dung beetles have demonstrated the capability to harbor parasites of public health concern, their preferences for pastures, forest floors, and other natural habitats, coupled with their species-specific dung removal patterns, could actually be of benefit in the removal of zoonotic parasites from the environment [102,103].

The included studies in this review were largely centered on filth flies, which feed and reproduce via human and animal fecal waste as well as through organic waste and garbage [6,7]. Similar to cockroaches, they are drawn to human food items where they may deposit parasitic organisms they have collected via external or internal contamination [1,7]. The mechanical transmission of ZEPs from these insect vectors in food preparation areas are a danger to health and safety in a variety of settings such as homes, restaurants, and hospitals. Food contamination from these insect vectors may be a neglected global threat to human and animal health. 

### 4.1. Protozoa

Many species of zoonotic protozoa were found naturally occurring within the insect vectors examined in the included titles. Additionally, experimental and mixed-methods study designs demonstrated additional vector potential for protozoal transmission. Cockroaches were found to be naturally contaminated with *Balantidium* spp. [59,68,80,81,95,97,99]. They also harbored the *Blastocystis* spp. [68,83,84,85,88,93,97,98,101]. One title discussed the presence of *Blastocystis* spp. in cockroaches, but the primary data were presented in a previous study not available in our search results [104,105]. Both cockroach and fly vectors were found to harbor the *Cryptosporidium* spp. (cockroach: [47,59,68,89,95,97,99]; fly: [54,58,60,61,64,67,73,82,100]). However, dung beetles were only infected experimentally [50,71]. *Entamoeba* spp. were also found in cockroaches and flies (cockroach: [59,62,68,80,81,87,88,89,95,97,99]; fly: [61,64,73,78,84,93]). Contamination with *Giardia* spp. among flies and cockroaches were common in the included publications (fly: [33,51,54,58,60,61,64,73,78,82,84,100]; cockroach: [80,88,89]). Oocysts from *Sarcocystis* spp. protozoal parasites were found in cockroaches and flies (cockroach: [34]; fly: [36]). *Toxoplasma gondii* was found in cockroaches, flies, and dung beetles, but only through experimental infection (fly: [22,24,26]; dung beetle: [43]; cockroach: [28,31,34,46]).

### 4.2. Cestodes

The insect vectors were found to be naturally contaminated with parasitic worms from the Cestoda class. Flies and cockroaches were found to have naive infection with *Hymenolepis* spp. (fly: [48,61,64,72,73,78]; cockroach: [76,89]). *Taenia* spp. were reported in flies, cockroaches, and dung beetles (fly: [44,48,61,64,73,74,78]; cockroach: [62,68,76,80,95,97]; dung beetle: [94]). Experimental studies showed that flies were also able to harbor *Echinococcus* spp. [20,21,90]. This may have also been true for cockroaches [89]. Moreover, a cockroach was experimentally infected with the *D. caninum* and *Mesocestoides* spp. [30]. 

### 4.3. Nematodes

The included studies most frequently found parasitic roundworms naturally present in the insect vectors. *Ascaris* spp. were reported in cockroaches and flies (cockroach: [10,18,59,68,80,81,83,88,89,97]; fly: [33,40,41,42,44,48,53,61,62,64,72,73,74,78,92]). In addition, flies and cockroaches were found with *Capillaria* spp. infection (fly: [44,48,53]; cockroach: [10]). Pinworm, or *E. vermicularis*, and other Oxyuridae spp. were found naturally occurring in cockroaches and flies (cockroach: [56,59,62,83,89]; fly: [53,72,78]). Cockroaches were also experimentally infected with the rat pinworm *S. obvelata* [30]. Dung beetles were reported to carry *Gongylonema* spp. [66]. Intestinal hookworms were discovered inside or on the outside of flies and cockroaches (fly: [33,40,41,44,61,64,72,73]; cockroach: [10,76,89,97]). Cockroaches had naïve infections with *Physaloptera* spp. and *Spiruroidea* spp. [95]. *Strongyloides* spp. and *Strongyloides*-like nematodes spp. were reported in flies and cockroaches (fly: [42,61,64,73]; cockroach: [59,68,80,89,97]). Fly and cockroach vectors were also harboring *Toxocara* spp. (fly: [42,44,48,53,77,84,91]; cockroach; [10,88,97]). Natural cockroach infection with *Trichinella* spp. was reported in the included studies [56]. Additionally, natural *Trichostrongylidae* spp. infection was reported in flies and cockroaches (fly: [53]; cockroach: [88]). *Trichuris* spp. was also found in fly and cockroach vectors (fly: [33,40,41,44,48,53,61,64,72,73,74,78,91]; cockroach: [10,59,62,68,80,81,88,89,97]).

### 4.4. Acanthocephala and Pentastomida

Both cockroach and fly vectors were found to be naturally infected with the *Acanthocephala* spp. (fly: [53]; cockroach: [32,95]). Moreover, cockroaches demonstrated natural infection with *Pentastomida* spp. [95]. 

### 4.5. Parasites of Potential Zoonotic Concern

Within the included studies, several species of enteric parasites that were investigated have a possible, or even probable, zoonotic transmission risk. They include the *Cyclospora* spp., which were found to be naturally occurring in cockroaches and flies (cockroach: [68,81,83,88,97,99]; fly: [84]). *O. dentatum* and *T. suis* were found in fly samples [92]. Dung beetles were naturally contaminated with *T. hydatigena* [94]. Additional experimental infection of the insect vectors with *Metastrongylus* spp., *P. turgida*, *S. equina*, *S. ransomi*, and *T. leonina* also yielded positive results [30,65].

### 4.6. Non-Pathogenic and Non-Zoonotic Organisms

In addition to the pathogenic agents found in the vectors, several of the included studies found non-pathogenic protozoa and flagellate. These organisms often indicate that the vector has had fecal exposure. *Entamoeba coli* (Grassi, 1879) was found in cockroaches, flies, and dung beetles [23,33,61,62,64,68,73,78,84,88,93,97]. *Entamoeba hartmanni* (Prowazek, 1912) was listed in a cockroach study [93]. *Iodamoeba bütschlii* (Prowazek, 1912) was also found in flies and cockroaches [68,78,88,93,97]. *Endolimax* *nana* (Wenyon and O’Connor, 1817) was found in dung beetles, cockroaches, and flies [23,68,84,88,93,97]. Cockroaches demonstrated naïve infection with the flagellate *Chilomastix mesnili* (Wenyon, 1910) [68,88,97]. 

Using the term zoonoses defined as diseases transmitted between humans and vertebrate animals, several pathogens that were found in the insect vectors but do not cause human infection or disease were excluded from the results table [106]. Those included *Cystoisopora* and *Isospora* spp., *Gregarina* spp., *Hydatigera* (*Taenia*) *taeniaeformis* (Batsch, 1786), *Hammerschmidtiella diesigni* (Hammerschmidt, 1838), *Lophomonas battaturm* (Stein, 1860), *Nyctotherus* spp., *Pharyngodon* spp., and *Thelastoma* spp. [30,34,43,83,87,93,95,97,99,101]. The inclusion criteria also required that the mode of transmission for the parasite be gastrointestinal, so that it could be considered an enteric parasite. This also excluded *Ascaridia galli* (Schrank, 1758), *Leptomonas* spp., *Pentatrichomonas* spp., and extraintestinal hookworm such as *Ancylostoma caninum* (Ercolani, 1859) and *Uncinaria* spp. [30,42,83,91,93]. Further investigation into the potential role these organisms have in the global parasitic burden of humans and animals is warranted. 

### 4.7. Sampling Locations and Risk Factors for Exposure

The insect vectors analyzed in the included studies originated from natural environments or were reared in laboratory settings. Overall, fly and cockroach insect vectors were collected from farms, pastures, open fields, and nearby livestock housing (i.e., barns) [39,40,51,52,54,57,58,60,65,67,69,78,82,90,92,95,100]. Fly samples were also drawn from village areas or areas of human habitation such as near kitchens, hospitals, food markets, and schools [27,33,38,40,41,42,43,44,54,60,61,64,69,72,73,77,78,100]. Nevertheless, many fly samples were collected near areas with a high risk of environmental contamination such as slaughterhouses/butchers and abattoirs, open defecation sites, and waste disposal or wastewater treatment areas [33,37,40,48,53,54,57,58,61,64,67,69,70,72,73,74,77,84,90]. Some fly specimens were also sampled from areas of public transportation, dog kennels, and from a zoo [33,36,53,91]. 

Cockroach specimens were also gathered from villages or household settings or human habitats [10,18,32,37,47,55,56,59,62,63,68,72,76,80,81,83,85,87,88,89,97,99,101]. Cockroach samples were also collected from a zoo and a pet store [95,98]. Dung beetles were sampled from wild settings of farms, pastures, forests, and fields [23,50,66,79]. However, one study did examine dung beetles in a village area [94]. Many studies used laboratory insect specimens for their analysis of parasite exposure and vector competence [19,20,21,22,24,25,26,27,28,29,30,34,39,43,45,46,49,57,65,75,86,96].

The authors of the studies identified water, sanitation, and hygiene-related risk factors that were associated with parasite presence in insect vectors, or were likely to increase the potential for parasite exposure and transmission. Inadequate or unsafe drinking water and sanitation services, infrastructure, and behaviors across individual, household, and community levels may contribute to the spread of ZEPs due to contact with, or food contamination from, flies and cockroaches [10,18,19,21,23,33,37,38,40,41,42,44,52,54,59,61,64,70,72,73,74,75,78,81,82,89,100]. Within the larger environment where a household is located, such as within a neighborhood, village, or municipality, potential drivers of ZEP transmission from insect vectors can result from open animal slaughterhouses, garbage and domestic waste piling up without regular removal, overcrowding, and insufficient or unsafe housing structures [35,38,40,41,42,44,47,52,54,55,58,64,67,70,72,73,74,76,78,81,82,84,88,89,91,100]. In particular, unmanaged, improperly stored, or untreated human waste within our living spaces, such as open defecation sites, may spread zoonotic enteric parasites through insect vectors [10,18,23,40,43,44,61,64,69,72,74,81]. Additionally, animal waste near human habitats is also a likely driver of ZEP transmission from insect vectors as they are contaminated by their contact with the human or animal waste for feeding and breeding [10,26,28,31,36,40,43,44,46,50,60,66,74,81,94,100]. Animal-related activities and husbandry in general could serve as a source of contamination for insects and people nearby as well as the animals themselves [10,18,20,21,26,28,42,44,46,47,50,52,53,57,60,65,66,90,91,94,98,100].

Several of studies mentioned that seasonality and environmental conditions such as rainfall, heat, and humidity could also contribute to the proliferation of the insect vectors and therefore increase the risk of exposure to ZEPs by humans and animals [18,28,29,38,39,40,41,62,69,71,82,90,91,97]. Moreover, the specific vector feeding, breeding, and habitat preferences coupled with their food predilections could also increase the risk of ZEP transmission [19,29,30,31,34,35,39,43,46,49,52,54,55,57,59,60,61,64,71,72,73,76,79,85,86,88,90,97,101]. The movements and behaviors of the insects should be considered, especially regarding food contamination. Unsafe food storage, preparation, and sale or service can transmit ZEPs to people and animals after contamination from a vector such as flies or cockroaches [19,20,21,26,29,33,37,38,40,46,54,55,57,62,63,68,69,72,73,81,85,90,101]. Furthermore, using insects as a food source for humans or animals, whether purposely or accidentally, can also present the risk of ZEP exposure [25,30,34,56,66,72,75,95,96].

### 4.8. Recommendations

One Health studies that simultaneously investigate parasite presence in humans, animals, food, and environmental reservoirs and vectors can demonstrate which groups and exposure pathways may be the biggest threat. For example, a recent publication conducted by a member of this research team found the zoonotic enteric parasites *Cryptosporidium* spp. and *Giardia* spp. among human, animals, flies, and drinking water in households in Mongolia [100]. The highest prevalence rate was round in the fly vectors (14.8%). This information, coupled with a household risk factor survey, demonstrated an association between ZEP presence and unimproved drinking water, not having a handwashing site at the home, domestic animal ownership, and rural location [100]. Researchers Dehghani and Kassiri even presented a question regarding the possible role of flies and cockroaches in the ongoing COVID-19 (SARS-CoV-2) pandemic due to their potential for environmental contamination [107]. More holistic research into water, sanitation, and hygiene (WASH) services and behaviors as well as food safety in personal and community spaces in connection with the prevalence of zoonotic enteric parasites in people, animals, and insect vectors who share these environments can shed light on how and where exposures are occurring. Armed with more robust One Health contexts for ZEP transmission routes, public and veterinary health professionals can collaborate with community members on targeted prevention and control efforts.

### 4.9. Limitations

This review identified studies of ZEPs in cockroaches, filth flies, and dung beetles from all over the world, yet due to the authors’ language barriers and lack of qualified translators, only English titles had the full text assessed. English abstracts from several titles illustrated parasite prevalence in vectors of interest and when possible, were included in the final analysis. However, the authors believe that valuable and important work in this subject area is likely to be available in additional languages and found through searching supplemental databases and sources. Furthermore, it is likely that titles of importance were left out of the results due to our search and screening parameters. For example, in one title, the authors spoke of a ZEP in cockroaches but referenced the initial presence data from another source that did not appear in our database results [104,105]. 

The breadth of parasites analyzed in the included studies demonstrate a wide range of species and hosts. In an effort to outline each pathogen, epidemiological details associated with every parasite were omitted. Information on exposure pathways and disease presentation associated with these zoonotic diseases would be helpful for public health professionals, veterinarians, and medical entomologists tasked with using this review for action against ZEP transmission. Similarly, validated information on the current systematic taxa of the pathogens included in the studies could be of further assistance in understanding more about these zoonotic enteric parasites.

## 5. Conclusions

One Health research collaboration is needed to build a better global assessment of ZEPs in insect vectors and the risks posed to human, animal, and environmental health. Implementing a joint approach to tackle these complex exposure pathways using experts and stakeholders in the disciplines of public health, epidemiology, veterinary sciences, biology, medical entomology, environmental health, and more can lead to targeted public and veterinary health education messages for the prevention and control of zoonotic enteric parasites. 

## Figures and Tables

**Table 1:** Characteristics of included studies examining zoonotic enteric parasites of public health concern in flies, cockroaches, and/or dung beetles.

Zoonotic Enteric Parasite(s) ^†^		Vector(s)	Country of Study	Sample Source	Type of Iinfection	Citation
Class and Family	*Genus* an/or *Species* and Natural Prevalence (%), When Provided					
Chromadorea, Ascarididae	*Ascaris* spp.	Cockroach	India	Village area	Experimental	Chandler 1926 [18]
Chromadorea Ancylostomatidae	Hookworm (Unspecified)					
Enoplea, Trichuridae	*Trichuris* spp.					
Lobosa, Entamoebidae	*Entamoeba histolytica*	Fly	England	Laboratory	Experimental	Roberts 1947 [19]
Cestoda, Taeniidae	*Echinococcus* spp.	Fly	United States	Laboratory	Experimental	Schiller 1954 [20]
Cestoda, Taeniidae	*Echinococcus granulosus*	Fly	South Africa	Laboratory	Experimental	Heinz and Brauns 1955 [21]
Conoidasida, Sarcocystidae	*Toxoplasma gondii*	Fly	Netherlands	Laboratory	Experimental	Laarman 1956 [22]
Chromadorea, Ascarididae	*Ascaris lumbricoides*	Dung beetle	United States	Farm/Field	Experimental	Miller et al. 1961 [23]
Zoomastigophora, Hexamitidae	*Giardia lamblia*					
Chromadorea Ancylostomatidae	Hookworm (*Necator americanus*)					
Enoplea, Trichuridae	*Trichuris trichiura*					
Conoidasida, Sarcocystidae	*T. gondii*	Fly	Brazil	Laboratory	Experimental	Paim and Queiroz 1963 * [24]
Chromadorea, Toxocaridae	*Toxocara canis*	Fly	England	Laboratory	Experimental	Pegg 1971 [25]
Conoidasida, Sarcocystidae	*T. gondii*	Fly	United States	Laboratory	Experimental	Wallace 1971 [26]
Chromadorea, Ascarididae	*Ascaris* spp.	Fly	Azerbaijan	Laboratory Village area	Mixed	Nadzhafov 1972 * [27]
Chromadorea Ancylostomatidae	Hookworm (Unspecified)					
Cestoda, Hymenolepididae	*Hymenolepis nana*					
Enoplea, Trichuridae	*Trichuris* spp.					
Conoidasida, Sarcocystidae	*T. gondii*	Cockroach	United States	Laboratory	Experimental	Wallace 1972 [28]
Enoplea, Trichinellidae	*Trichinella spiralis*	Cockroach	United States	Laboratory	Experimental	Young and Babero 1974 [29]
Chromadorea, Ascarididae	*Ascaris columnaris* (*Baylisascaris procyonis*)	Cockroach	United States	Laboratory	Experimental	Young 1975 [30]
	*Ascaris suum*					
Cestoda, Dipylidiidae	*Dipylidium caninum*					
Cestoda, Hymenolepididae	*Hymenolepis dimenuta*					
	*H. nana*					
Cestoda, Mesocestoididae	*Mesocestoides lineatus*					
Chromadorea, Physalopteridae	*Physaloptera turgida* ^§^					
Chromadorea, Setariidae	*Setaria equina* ^§^					
Chromadorea, Oxyuridae	*Syphacia obvelata*					
Chromadorea, Toxocaridae	*Toxascaris leonine* ^§^					
	*T. canis*					
	*Toxocara cati*					
Conoidasida, Sarcocystidae	*T. gondii*	Cockroach	Costa Rica	Unspecified	Experimental	Chinchilla and Ruiz 1976 [31]
Lobosa, Entamoebidae	*Entamoeba* spp.	Cockroach	Tunisia	Urban area	Natural	Gonzalez and Mishra 1976 * [32]
Archiacanthocephala, Moniliformidae	*Moniliformis moniliformis*					
Chromadorea, Ascarididae	*A. lumbricoides* (18.8–62.5%)	Fly	Bangladesh	Abattoir/Butchery/Slaughterhouse/ Food market Hospital Household Open defecation area Public transportation Waste disposal area	Natural	Khan and Huq 1978 [33]
Zoomastigophora, Hexamitidae	*Giardia* spp. (6.2%)					
Chromadorea Ancylostomatidae	Hookworm (Unspecified; 15.6%)					
Enoplea, Trichuridae	*Trichuris trichiura* (46.9%)					
Conoidasida, Sarcocystidae	*Sarcocystis muris*	Cockroach	United States	Laboratory	Experimental	Smith and Frenkel 1978 [34]
	*T. gondii*					
Cestoda, Taeniidae	*Taenia saginata*	Dung beetle	Poland	Unspecified	Experimental	Lonc 1980 [35]
Conoidasida, Sarcocystidae	*Sarcocystis* spp.	Fly	England	Dog Kennel	Natural	Markus 1980 [36]
Zoomastigophora, Hexamitidae	*Giardia intestinalis*	Cockroach Fly	Poland	Open defecation area Waste disposal area	Mixed	Kasprzak and Majewska 1981 * [37]
Chromadorea, Ascarididae	*A. lumbricoides*	Fly	Nigeria	School/University	Experimental	Dipeolu 1982 [38]
Chromadorea Ancylostomatidae	Hookworm (Unspecified)					
Cestoda, Taeniidae	*Taenia hydatigena* ^§^	Fly	New Zealand	Farm/Field Laboratory	Mixed	Lawson and Gemmell 1985 [39]
Chromadorea, Ascarididae	*A. lumbricoides*	Fly	Malaysia	Farm/Field Household Waste disposal area	Natural	Sulaiman et al. 1988 [40]
Chromadorea Ancylostomatidae	Hookworm (*Necator americanus*)					
Enoplea, Trichuridae	*T. trichiura*					
Chromadorea, Ascarididae	*A. lumbricoides*	Fly	Malaysia	Household	Natural	Sulaiman et al. 1989 [41]
Chromadorea Ancylostomatidae	Hookworm (*Necator americanus* and/or *Ancylostoma duodenale* ^§^)					
Enoplea, Trichuridae	*T. trichiura*					
Chromadorea, Ascarididae	*A. lumbricoides* (0.20–0.81%)	Fly	Nigeria	Food market Household	Natural	Umeche and Mandah 1989 [42]
Chromadorea, Strongyloididae	*Strongyloides stercoralis* (0.40–1.80%)					
Chromadorea, Toxocaridae	*T. canis* (2.40–2.11%)					
Conoidasida, Sarcocystidae	*T. gondii*	Dung beetle	Japan	Laboratory School/University	Mixed	Saitoh and Itagaki 1990 [43]
Chromadorea, Ascarididae	*Ascaris* spp. (72.8–82.4%)	Fly	Philippines	Urban area	Natural	Monzon et al. 1991 [44]
Enoplea, Capillariidae	*Capillaria hepatica* (0.0–0.005%)					
Chromadorea Ancylostomatidae	Hookworm (Unspecified; (0.02–13.1%)					
Cestoda, Taeniidae	*Taenia* spp. (0.005–0.02%)					
Chromadorea, Toxocaridae	*Toxocara* spp. (0.005–0.04%)					
Enoplea, Trichuridae	*T. trichiura* (18.8–60.1%)					
Archiacanthocephala, Moniliformidae	*M. moniliformis*	Cockroach	Scotland	Laboratory	Experimental	Stoddart et al. 1991 [45]
Conoidasida, Sarcocystidae	*T. gondii*	Cockroach	Costa Rica	Laboratory	Experimental	Chinchilla et al. 1994 [46]
Conoidasida, Cryptosporidiidae	*Cryptosporidium* spp.	Cockroach	Peru	Garden Household	Natural	Zerpa and Huicho 1994 [47]
Chromadorea, Ascarididae	*Ascaris* spp.	Fly	Slovakia	Wastewater treatment area	Natural	Juris et al. 1995 * [48]
Enoplea, Capillariidae	*Capillaria* spp.					
Cestoda, Hymenolepididae	*Hymenolepis* spp.					
Cestoda, Taeniidae	*Taenia* spp.					
Chromadorea, Toxocaridae	*Toxocara* spp.					
Enoplea, Trichuridae	*Trichuris* spp.					
Conoidasida, Cryptosporidiidae	*Cryptosporidium parvum*	Fly	United States	Laboratory	Experimental	Graczyk et al. 1999 [49]
Conoidasida, Cryptosporidiidae	*C. parvum*	Dung beetle	Czech Republic	Farm/Field Forest	Experimental	Mathison and Ditrich 1999 [50]
Zoomastigophora, Hexamitidae	*G. lamblia* (22%)	Fly	Spain	Farm/Field	Natural	Doiz et al. 2000 [51]
Conoidasida, Cryptosporidiidae	*C. parvum*	Fly	United States	Farm/Field	Mixed	Graczyk et al. 2000 [52]
Palaeacanthocephala, Unspecified	*Acanthocephala* spp.	Fly	Brazil	Waste disposal area Zoo	Natural	de Oliveira et al. 2002 * [53]
Chromadorea, Ascarididae.	*Ascaris* spp.					
Enoplea, Capillariidae	*Capillaria* spp.					
Chromadorea, Ascarididae.	*Toxascaris* spp. ^§^					
Chromadorea, Toxocaridae	*Toxocara* spp.					
Chromadorea, Trichostrongylidae	*Trichostrongylidae* spp.					
Enoplea, Trichuridae	*Trichuris* spp.					
Chromadorea, Oxyuridae	*Unspecified oxyuridae* spp.					
Conoidasida, Cryptosporidiidae	*C. parvum*	Fly	United States	Farm/Field Food market Waste disposal area	Natural	Graczyk et al. 2003 [54]
Zoomastigophora, Hexamitidae	*G. lamblia*					
Lobosa, Entamoebidae	*Entamoeba histolytica/dispar* (10.3–25.4%)	Cockroach	Taiwan	Kitchen area Laboratory School/University	Mixed	Pai et al. 2003 [55]
Chromadorea, Oxyuridae	*Enterobius vermicularis* (3%)	Cockroach	United States	Hospital School/University	Natural	Chan et al. 2004 [56]
Enoplea, Trichinellidae	*Trichinella* spp. (1.0%)					
Conoidasida, Cryptosporidiidae	*C. parvum*	Fly	United States Poland	Farm/Field Laboratory Waste disposal area	Mixed	Graczyk et al. 2004 [57]
Conoidasida, Cryptosporidiidae	*C. parvum*	Fly	Poland	Farm/Field Waste disposal area	Natural	Szostakowska et al. 2004 [58]
Zoomastigophora, Hexamitidae	*G. lamblia*					
Chromadorea, Ascarididae	*A. lumbricoides*	Cockroach	Nigeria	Household	Natural	Tatfeng et al. 2005 [59]
Litostomatea, Balantiididae	*Balantidium coli*					
Conoidasida, Cryptosporidiidae	*C. parvum*					
Lobosa, Entamoebidae	*E. histolytica*					
Chromadorea, Oxyuridae	*E. vermicularis*					
Chromadorea, Strongyloididae	*S. stercoralis*					
Enoplea, Trichuridae	*T. trichiura*					
Conoidasida, Cryptosporidiidae	*Cryptosporidium* spp. (55.56%)	Fly	United States	Farm/Field Garden School/University	Natural	Conn et al. 2007 [60]
Zoomastigophora, Hexamitidae	*Giardia* spp. (7.94%)					
Chromadorea, Ascarididae	*A. lumbricoides*	Fly	Ethiopia	Abattoir/Butchery/Slaughterhouse/ Food market Open defecation area Waste disposal area	Natural	Getachew et al. 2007 [61]
Conoidasida, Cryptosporidiidae	*Cryptosporidium* spp.					
Lobosa, Entamoebidae	*E. histolytica*/*dispar*					
Zoomastigophora, Hexamitidae	*G. lamblia*					
Chromadorea Ancylostomatidae	Hookworm (Unspecified)					
Cestoda, Hymenolepididae	*H. nana*					
Chromadorea, Strongyloididae	*S. stercoralis*					
Cestoda, Taeniidae	*Taenia* spp.					
Enoplea, Trichuridae	*T. trichiura*					
Chromadorea, Ascarididae	*A. lumbricoides*	Cockroach	Ethiopia	Household	Natural	Kinfu and Erko 2008 [62]
Lobosa, Entamoebidae	*E. histolytica*/*dispar*					
Chromadorea, Oxyuridae	*E. vermicularis*					
Cestoda, Taeniidae	*Taenia* spp.					
Enoplea, Trichuridae	*T. trichiura*					
Chromadorea, Toxocaridae	*T. canis*	Cockroach	India	Kitchen area	Experimental	Sasmal et al. 2008 [63]
Chromadorea, Ascarididae	*A. lumbricoides* (36.9%)	Fly	Ethiopia	Abattoir/Butchery/Slaughterhouse/ Food market Waste disposal area	Natural	Fetene and Worku 2009 [64]
Conoidasida, Cryptosporidiidae	*Cryptosporidium* spp. (16.7%)					
Lobosa, Entamoebidae	*E. histolytica/dispar* (48.1%)					
Zoomastigophora, Hexamitidae	*G. lamblia* (10.4%)					
Chromadorea Ancylostomatidae	Hookworm (Unspecified; 13.0%)					
Cestoda, Hymenolepididae	*H. nana* (0.6%)					
Chromadorea, Strongyloididae	*S. stercoralis* (1.7%)					
Cestoda, Taeniidae	*Taenia* spp. (8.4%)					
Enoplea, Trichuridae	*T. trichiura* (38.8%)					
Chromadorea, Ascarididae	*A. suum*	Fly	Germany	Farm/Field Laboratory	Mixed	Förster et al. 2009 [65]
Chromadorea, Metastrongylidae	*Metastrongylus* spp. ^§^					
Chromadorea, Strongyloididae	*Strongyloides ransomi* ^§^					
Enoplea, Trichuridae	*Trichuris suis* ^§^					
Chromadorea, Gongylomatidae	*Gongylonema* spp. (17.7%)					
Chromadorea, Rhabditidae	*Rhabditis* spp. (2.2%)					
Chromadorea, Gongylomatidae	*Gongylonema* spp. (17.7%)	Dung beetle	Iran	Farm/Field	Natural	Mowlavi et al. 2009 [66]
Chromadorea, Rhabditidae	*Rhabditis* spp. (2.2%)					
Conoidasida, Cryptosporidiidae	*Cryptosporidium* spp. (18.9%)	Fly	Poland	Farm/Field Waste disposal area	Natural	Racewicz et al. 2009 * [67]
Chromadorea, Ascarididae	*A. lumbricoides* (0.3%)	Cockroach	Thailand	Food market	Natural	Chamavit et al. 2011 [68]
Litostomatea, Balantiididae	*B. coli* (5.8%)					
Bigyra, Blastocystidae	*Blastocystis hominis* (1.2%)					
Conoidasida, Cryptosporidiidae	*Cryptosporidium* spp. (28.1%)					
Conoidasida, Eimeriidae	*Cyclospora* spp. (1.3%) ^§^					
Lobosa, Entamoebidae	*E. histolytica*/*dispar* (4.6%)					
Chromadorea, Strongyloididae	*S. stercoralis* (0.8%)					
Cestoda, Taeniidae	*Taenia* spp. (0.1%)					
Enoplea, Trichuridae	*T. trichiura* (0.3%)					
Conoidasida, Cryptosporidiidae	*Cryptosporidium* spp.	Fly	Ethiopia	Abattoir/Butchery/Slaughterhouse/ Farm/Field Food market Open defecation area	Mixed	Fetene et al. 2011 [69]
Unspecified	Unspecified helminths and protozoa	Fly	Brazil	Waste disposal area	Natural	Ribeiro et al. 2011 * [70]
Conoidasida, Cryptosporidiidae	*Cryptosporidium* spp.	Dung beetle	Australia	Unspecified	Experimental	Ryan et al. 2011 [71]
Chromadorea, Ascarididae	Flies *Ascaris* spp.	Fly Cockroach	Egypt	Household Open defecation area	Natural	El-Sherbini and Gneidy 2012 [72]
Chromadorea, Oxyuridae	*E. vermicularis*					
Chromadorea Ancylostomatidae	Hookworm (Unspecified)					
Cestoda, Hymenolepididae	*H. nana*					
Enoplea, Trichuridae	*T. trichiura*					
	Cockroaches Unspecified parasitic agents					
Chromadorea, Ascarididae	*A. lumbricoides* (34.08%)	Fly	Nigeria	Abattoir/Butchery/Slaughterhouse/ Food market Open defecation area Waste disposal area	Natural	Adenusi and Adewoga 2013 [73]
Conoidasida, Cryptosporidiidae	*Cryptosporidium* spp. (1.81%)					
Lobosa, Entamoebidae	*E. histolytica*/*dispar* (27.26%)					
Zoomastigophora, Hexamitidae	*G. lamblia* (3.34%)					
Chromadorea Ancylostomatidae	Hookworm (Unspecified; 20.45%)					
Cestoda, Hymenolepididae	*H. nana* (1.11%)					
Chromadorea, Strongyloididae	*S. stercoralis* (3.89%)					
Cestoda, Taeniidae	*Taenia* spp. (2.36%)					
Enoplea, Trichuridae	*T. trichiura* (25.87%)					
Chromadorea, Ascarididae	*A. lumbricoides* (52.2%)	Fly	Nigeria	Open defecation area Waste disposal area	Natural	Adenusi and Adewoga 2013 [74]
Cestoda, Taeniidae	*Taenia* spp. (1.0%)					
Enoplea, Trichuridae	*T. trichiura* (47.2%)					
Chromadorea, Ascarididae	*A. suum*	Fly	Sweden	Laboratory	Experimental	Lalander et al. 2013 [75]
Chromadorea Ancylostomatidae	Hookworm (*Ancylostoma duodenale*) (4.9%) ^§^	Cockroach	Ghana	Hospital	Natural	Tetteh-Quarcoo et al. 2013 [76]
Cestoda, Hymenolepididae	*H. nana* (1.6%)					
Cestoda, Taeniidae	*Taenia* spp. (1.6%)					
Chromadorea, Toxocaridae	*Toxocara* spp.	Fly	Thailand	Farm/Field Food market School/University Waste disposal area	Natural	Bunchu et al. 2014 [77]
Chromadorea, Ascarididae	*Ascaris* spp.	Fly	Brazil	Farm/Field School/University	Natural	Cruz Souza Lima et al. 2014 [78]
Lobosa, Entamoebidae	*Entamoeba* spp.					
Chromadorea, Oxyuridae	*E. vermicularis*					
Zoomastigophora, Hexamitidae	*Giardia* spp.					
Cestoda, Hymenolepididae	*H. nana*					
Cestoda, Taeniidae	*Taenia* spp.					
Enoplea, Trichuridae	*Trichuris* spp.					
Cestoda, Taeniidae	*Taenia solium*	Dung beetle	Peru	Farm/Field	Experimental	Gomez-Puerta et al. 2014 [79]
Chromadorea, Ascarididae	*A. lumbricoides*	Cockroach	Ethiopia	Household	Natural	Hamu et al. 2014 [80]
Litostomatea, Balantiididae	*B. coli*					
Lobosa, Entamoebidae	*Entamoeba* spp.					
Zoomastigophora, Hexamitidae	*G. duodenalis*					
Cestoda, Taeniidae	*Taenia* spp.					
Enoplea, Trichuridae	*T. trichiura*					
	Unspecified *Strongyloides*-like nematodes					
Chromadorea, Ascarididae	*A. lumbricoides* (2.9–13.2%)	Cockroach	Nigeria	Household	Natural	Isaac et al. 2014 [81]
Litostomatea, Balantiididae	*B. coli* (1.1–1.2%)					
Lobosa, Entamoebidae	*E. histolytica* (1.2–2.2%)					
Enoplea, Trichuridae	*T. trichiura* (4.4–4.7%)					
	*Unspecified coccidia* spp. (3.3%) ^§^					
Conoidasida, Cryptosporidiidae	*Cryptosporidium* spp.	Fly	China	Farm/Field	Natural	Zhao et al. 2014 [82]
Zoomastigophora, Hexamitidae	*Giardia* spp.					
Chromadorea, Ascarididae	*Ascaris* spp.	Cockroach	Venezuela	Food market Hospital School/University	Natural	Cazorla Perfetti et al. 2015 * [83]
Bigyra, Blastocystidae	*Blastocystis* spp. (82.9%)					
Conoidasida, Eimeriidae	*Cyclospora* spp. ^§^					
Chromadorea, Oxyuridae	*E. vermicularis*					
Bigyra, Blastocystidae	*Blastocystis* spp.	Fly	Venezuela	Waste disposal area	Natural	Muñoz 2015 * [84]
Conoidasida, Eimeriidae	*Cyclospora cayetanensis* ^§^					
Lobosa, Entamoebidae	*E. histolytica*					
Zoomastigophora, Hexamitidae	*G. intestinalis*					
Chromadorea, Toxocaridae	*Toxocara* spp.					
Chromadorea, Ascarididae	*Ascaris* spp. (33.76%)	Cockroach	Cameroon	Household	Natural	Atiokeng Tatang et al. 2017 [10]
Enoplea, Capillariidae	*Capillaria* spp. (6.16%)					
Chromadorea Ancylostomatidae	Hookworm (Unspecified; 4.86%)					
Chromadorea, Toxocaridae	*Toxocara* spp. (4.86%)					
Enoplea, Trichuridae	*T. trichiura* (11.97%)					
Bigyra, Blastocystidae	*Blastocystis* spp. (40.4%)	Cockroach	Malaysia	Food market Household Waste disposal area	Natural	Farah et al. 2017 [85]
Chromadorea, Toxocaridae	*T. canis*	Cockroach	Mexico	Laboratory	Experimental	González-García et al. 2017 [86]
Unspecified	*Amoeba* spp. (25.4%)	Cockroach	Spain	Hospital Kitchen area School/University	Natural	Martínez-Girón et al. 2017 [87]
Chromadorea, Ascarididae	*A. lumbricoides* (3%)	Cockroach	Turkey	Household	Natural	Oğuz et al. 2017 [88]
Bigyra, Blastocystidae	*B. hominis* (41%)					
Lobosa, Entamoebidae	*E. histolytica*/*dispar* (16.7%)					
Zoomastigophora, Hexamitidae	*Giardia* spp. (13.6%)					
Chromadorea, Toxocaridae	*Toxocara* spp. (3%)					
Chromadorea, Trichostrongylidae	*Trichostrongylus* spp. (1.5%)					
Enoplea, Trichuridae	*T. trichiura* (1.5%)					
	Unspecified unsporulated coccidial oocyst (7.6%) ^§^					
Chromadorea, Ascarididae	*A. lumbricoides* (61.3%)	Cockroach	Nigeria	Household Kitchen area	Natural	Adenusi et al. 2018 [89]
Conoidasida, Cryptosporidiidae	*Cryptosporidium* spp. (13.85)					
Lobosa, Entamoebidae	*E. histolytica*/*dispar* (44.1%)					
Chromadorea, Oxyuridae	*E. vermicularis* (17.2%)					
Zoomastigophora, Hexamitidae	*G. lamblia* (18.7%)					
Chromadorea Ancylostomatidae	Hookworm (Unspecified; 11.6%)					
Cestoda, Hymenolepididae	*H. nana* (11.6%)					
Chromadorea, Strongyloididae	*S. stercoralis* (11.7%)					
Cestoda, Taeniidae	*Taenia* spp./*Echinococcus* spp. (10.5%)					
Enoplea, Trichuridae	*T. trichiura* (55.8%)					
Cestoda, Taeniidae	*E. granulosus*	Fly	Iran	Abattoir/Butchery/Slaughterhouse/ Farm/Field	Mixed	Hemmati et al. 2018 [90]
Chromadorea, Toxocaridae	*T. canis*	Fly	Ukraine	Dog kennel	Natural	Paliy et al. 2018 [91]
Enoplea, Trichuridae	*Trichuris vulpis*					
Chromadorea, Ascarididae	*A. suum*	Fly	Ukraine	Farm/Field	Natural	Paliy et al. 2018 [92]
Chromadorea, Chabertiidae	*Oesophagostomum dentatum* ^§^					
Enoplea, Trichuridae	*T. suis* ^§^					
Bigyra, Blastocystidae	*Blastocystis* spp.	Fly	Venezuela	Unspecified	Natural	Valles et al. 2018 * [93]
Lobosa, Entamoebidae	*E. histolytica*/*dispar*					
Cestoda, Taeniidae	*T. hydatigena* ^§^	Dung beetle	Peru	Farm/Field Village area	Natural	Vargas-Calla et al. 2018 [94]
	*T. solium*					
Palaeacanthocephala, Unspecified	*Acanthocephala* spp. (0.67%)	Cockroach	Various	Farm/Field Pet store	Natural	Gałęcki and Sokół 2019 [95]
Litostomatea, Balantiididae	*Balantidium* spp. (4.67%)					
Conoidasida, Cryptosporidiidae	*Cryptosporidium* spp. (11.87%)					
Lobosa, Entamoebidae	*Entamoeba* spp. (4.53%)					
Chromadorea, Physalopteridae	*Physaloptera* spp. (3.07%)					
Cestoda, Unspecified	*Unspecified cysticercoids* (0.53%)					
Maxillopoda, Unspecified	*Unspecified pentastomida* spp. (0.67%)					
Chromadorea, Spiruridae	*Unspecified spiruroidea* spp. (1.87%)					
Chromadorea, Ascarididae	*A. suum*	Fly	Germany	Laboratory	Experimental	Muller et al. 2019 [96]
Chromadorea, Ascarididae	*A. lumbricoides* (5.9%)	Cockroach	Thailand	Food market	Natural	Dokmaikaw and Suntaravitun 2020 [97]
Litostomatea, Balantiididae	*B. coli* (1.1%)					
Bigyra, Blastocystidae	*B. hominis* (6.6%)					
Conoidasida, Cryptosporidiidae	*Cryptosporidium* spp. (15.4%)					
Conoidasida, Eimeriidae	*Cyclospora* spp. (7.0%) ^§^					
Lobosa, Entamoebidae	*E. histolytica*/*dispar* (8.5%)					
Chromadorea Ancylostomatidae	Hookworm (Unspecified; 2.2%)					
Chromadorea, Strongyloididae	*S. stercoralis* (4.4%)					
Cestoda, Taeniidae	*Taenia* spp. (5.1%)					
Chromadorea, Toxocaridae	*Toxocara* spp. (8.5%)					
Enoplea, Trichuridae	*T. trichiura* (6.3%)					
Bigyra, Blastocystidae	*Blastocystis* spp. (82.8%)	Cockroach	China	Zoo	Natural	Ma et al. 2020 [98]
Litostomatea, Balantiididae	*B. coli* (2.1%)	Cockroach	Spain	Household	Natural	van Woerden et al. 2020 [99]
Conoidasida, Cryptosporidiidae	*Cryptosporidium* spp. (9%)					
Lobosa, Entamoebidae	*Entamoeba* spp. (12.7%)					
	*Unspecified coccidia* spp. (8.4%) ^§^					
Conoidasida, Cryptosporidiidae	*Cryptosporidium* spp. (0.9%)	Fly	Mongolia	Household Kitchen area	Natural	Barnes et al. 2021 [100]
Zoomastigophora, Hexamitidae	*Giardia* spp. (14.8%)					
Bigyra, Blastocystidae	*Blastocystis* spp. (2.1%)	Cockroach	Iran	Hospital	Natural	Motevalli-Haghi et al. 2021 [101]

^†^ Only parasites with a primary enteric transmission route were included; ^§^ Unrecognized but potential zoonotic risk; * Table information from the article’s English abstract/summary only; The underline in Table 1 refers to a group of many different species of parasites.

**Table 2:** Risk factors for exposure to and/or transmission of zoonotic enteric parasites from flies, cockroaches, or dung beetles, as addressed in the included studies.

Risk Factor	Citations
Inadequate water and sanitation services or infrastructure at household or community level	[10,18,19,21,23,33,37,38,40,41,42,44,52,54,59,61,64,70,72,73,74,75,78,81,82,89,100]
Open defecation site near human or animal activities	[10,18,23,40,43,44,61,64,69,72,74,81]
Unmanaged animal waste near human or animal activities	[10,26,28,31,36,40,43,44,46,50,60,66,74,81,94,100]
Poor environmental hygiene, overcrowding, open slaughter, and/or a lack of garbage removal and processing services	[35,38,40,41,42,44,47,52,54,55,58,64,67,70,72,73,74,76,78,81,82,84,88,89,91,100]
Seasonality and environmental conditions for insect vector proliferation	[18,28,29,38,39,40,41,62,69,71,82,90,91,97]
Unsafe food preparation, storage, sale, and/or service	[19,20,21,26,29,33,38,40,46,54,55,57,62,63,68,69,72,73,81,85,90,101]
Insect vector feeding behaviors and preferences, movement patterns, and living habitat predilection	[19,29,30,31,34,35,39,43,46,49,52,54,55,57,59,60,61,64,71,72,73,76,79,85,86,88,90,97,101]
Animal contact, husbandry, and proximity to living spaces	[10,18,20,21,26,28,42,44,46,47,50,52,53,57,60,65,66,90,91,94,98,100]
Purposeful or accidental ingestion of contaminated insect vector by animals or humans	[25,30,34,56,66,72,75,95,96]

## Data Availability

All of the data used in this study are openly available.

## Supplementary Materials

The following supporting information can be downloaded at: https://www.mdpi.com/article/10.3390/pathogens11010090/s1, Table S1: Search Strings and Results by Database.
