# Supplementary material for: A Systematic Review of Zoonotic Enteric Parasites Carried by Flies, Cockroaches, and Dung Beetles
Source: Pathogens. 2022 Jan 13;11(1):90. doi: 10.3390/pathogens11010090 (PMC8778527; doi:10.3390/pathogens11010090)
Supplement: Supplementary file 1 [file pathogens-11-00090-s001.zip › pathogens-1534504-supplementary.pdf]

Table S1: Internal Protocol, Search Strings and Results by Database

Project Lead: Amber N. Barnes
Team Members: Avi Patel, Meg Jenkins, and Kelly Rhoden
Review Date: Initial Process Spring-Summer 2021; Final Search December 2021 for additional titles
Institution: University of North Florida

Research Question:
What is the role of insect vectors in the spread of zoonotic enteric parasites (ZEPs)?

Study Aims:
1. Determine which zoonotic enteric parasites (ZEPs) have been reported in filth flies, cockroaches, and dung beetles
2. Identify water, sanitation and hygiene risk factors that are associated with transmission or exposure in the different domains presented (i.e. household, school, farm, slaughterhouse, restaurant, hospital) etc.
3. Evaluate opportunities for One Health guidance, interventions or collaboration to prevent further spread of disease within these domains from the insect vectors

Inclusion Criteria:
- Peer-Reviewed Scholarly Journals
- Primary research documenting the presence of a ZEP in an insect vector
  - *Experimental*
  - *Natural infection*
- All Languages
  - *Non-English material became a criteria for exclusion when we realized our full-text results spanned numerous languages and we did not have access to institutional translation services*
  - *We were able to include non-English publications that had abstracts or summaries written in English that demonstrated our other inclusion criteria*
- All Years

Exclusion Criteria:
- Any publication that is not peer-reviewed
- Technical Reports, Book Chapters, etc.
- Broad inferences or discussions on the possibility or biological plausibility of ZEPs in insect vectors
- Parasite or disease is not transmitted through gastro-intestinal route
  - Leishmaniasis
  - “Sleeping sickness”
  - Trypanosomiasis
- Insect vector is not a filth fly, dung beetle, or cockroach
  - Mosquitoes
  - Sand Flies
  - Tsetse flies/tsetse
  - Glossina
  - Fruit flies
- Results that did not show the presence of the ZEP in the vector (null results)
- Non-English language
  - *This exclusion criteria was used during the full-text screening process only*

Screening Process:

1. Gather all available publications from the databases listed below
2. Keep track of every step of the screening process by using a spreadsheet with titles, their inclusion criteria, their exclusion criteria, etc.
3. Working in pairs, screen the titles and abstracts for eligibility
   - Any questionable or unsure titles should be retained
   - Titles that do not contain an abstract should be retained
4. Retrieve the full-text version of all titles that have not been excluded
5. Each accessible full-text manuscript should be read in its entirety to look for the inclusion criteria above
   - This will be done in pairs but each member will read the articles and provide their decision
   - If the title does not meet these qualifications, a specific reason should be given (ex. results of study did not show any ZEP in the vector)
   - If there is not consensus between the pair, the lead researcher (AB) will be the tie-breaker
6. A quality control check of the excluded titles will be done by AB before final inclusion of studies in analysis

Databases for Search:

ABI/INFORM Collection
Agricola
Agriculture Collection
Earth, Atmospheric, and Aquatic Sciences Collection
Environment Complete
Agricultural and Environmental Science Collection
Environmental Studies and Policy
Google Scholar
Health and Medicine
MEDLINE (Proquest)
Nursing and Allied Health Outcomes
Pubmed
Science Direct
TOXLINE
Web of Science Core Collection

Database	Results from First Search	Results from Final Search
**ABI/INFORM Collection**	**2/2/21 = 47**	**12/27/21 = 3**
**Search String:**<br>ab("Zoonotic enteric pathogen" OR "Zoonotic enteric pathogens" OR "Zoonotic enteric parasite" OR "Zoonotic enteric parasites" OR "Zoonotic parasite" OR "Zoonotic parasites" OR "Animal-to-Human parasite" OR "Animal-to-Human parasites" OR "Foodborne parasite" OR "Foodborne parasites" OR "Waterborne parasite" OR "Waterborne parasites" OR "Water-related parasite" OR "Water-related parasites" OR "Enteric parasite" OR "Enteric parasites" OR parasitism OR "Zoonotic intestinal helminth infection" OR "Zoonotic intestinal helminth infections" OR Helminthosis OR Helminthiasis OR Ascarosis OR Ascariasis OR Ancylostomosis OR Ancylostomiasis OR Trichuriosis OR Trichuriasis OR Strongyloidosis OR Strongyloidiasis OR Helminth* OR Ascaris OR Ancylostoma OR Hookworm* OR Trichuris OR Strongyloides OR Alaria OR "rat lungworm" OR "rat lungworms" OR "Echinostoma" OR "Lagochilascaris minor" OR "Zoonotic trypanosomosis" OR Trypanosomiasis OR Chagas OR "Trypanosoma cruzi" OR "Zoonotic intestinal protozoal infection" OR "Zoonotic intestinal protozoal infections" OR Protozoosis OR Protozoasis OR Giardiosis OR Giardiasis OR Cryptosporidiosis OR Blastocystosis OR Sarcocystosis OR Cyclosporiasis OR Cyclospora OR Amoebiasis OR "Amoebic dysentery" OR Entamoeba OR Balantidosis OR Protozoa OR		

<table>
<tr><td colspan="3">Giardia OR Cryptosporidium OR Blastocystis OR Sarcocystis OR "Cyclospora cayetanensis" OR tiab OR "Entamoeba histolytica" OR "Balantidium coli" OR Trichinellosis OR Trichinosis OR Trichinella OR Toxoplasmosis OR TORCH OR Toxoplasma OR Toxocarosis OR Toxocariasis OR Toxocariosis OR "Larva migrans" OR Toxocara OR Taeniosis OR Taeniasis OR Tapeworm OR Tapeworms OR Taenia OR "Foodborne trematodosis" OR trematodosis OR Trematodiasis OR Fasciolosis OR Fascioliosis OR Fasciolasis OR Fascioliasis OR Distomatosis OR Fasciolopsiasis OR Fasciolopsiosis OR Opisthorchosis OR Opisthorchiasis OR Clonorchiosis OR Clonorchiasis OR Paragonimosis OR Paragonimiasis OR Metagonimus OR Heterophyiasis OR Fluke OR Flukes OR Trematode OR Trematodes OR Fasciola OR Fasciolopsis OR Opisthorchis OR Clonorchis OR Paragonimus OR "Minute intestinal fluke" OR "Minute intestinal flukes" OR "Haplorchis pumilio" OR "Metagonimus yokogawai" OR "Heterophyes" OR Diphyllobothriosis OR Diphyllobothriasis OR Bothriocephalosis OR Bothriocephaliasis OR Diphyllobothrium OR Bothriocephalus OR "Broad tapeworm" OR "Broad tapeworms" OR "Fish tapeworm" OR "Fish tapeworms" OR Cysticercosis OR Neurocysticercosis OR "Taenia solium" OR "Cystic echinococcosis" OR "Hydatid disease" OR "Hydatid diseases" OR Hydatidosis OR "Echinococcus granulosus" OR "Hydatid cyst" OR "Hydatid cysts" OR "Alveolar echinococcosis" OR "Alveolar hydatidosis" OR "Echinococcus multilocularis") AND (fly OR flies OR “filth fly” OR “filth flies” OR Sarcophagidae OR Muscidae OR Calliphoridae OR “household fly” OR “households flies” OR “Musca Domestica” OR “synanthropic fly” OR “synanthropic flies” OR cockroach* OR “Blatella germanica” OR “dung beetle” OR “dung beetles” OR “copraphagic beetle” OR “Copraphagic beetles” OR Onthophagus)
*Limit to scholarly journals</td></tr>
<tr><th>Agricola</th><th>1/28/21 = 824</th><th>12/27/21 = 19</th></tr>
<tr><td colspan="3">Ab,ti(("Zoonotic enteric pathogen" OR "Zoonotic enteric pathogens" OR "Zoonotic enteric parasite" OR "Zoonotic enteric parasites" OR "Zoonotic parasite" OR "Zoonotic parasites" OR "Animal-to-Human parasite" OR "Animal-to-Human parasites" OR "Foodborne parasite" OR "Foodborne parasites" OR "Waterborne parasite" OR "Waterborne parasites" OR "Water-related parasite" OR "Water-related parasites" OR "Enteric parasite" OR "Enteric parasites" OR parasitism OR "Zoonotic intestinal helminth infection" OR "Zoonotic intestinal helminth infections" OR Helminthosis OR Helminthiasis OR Ascarosis OR Ascariasis OR Ancylostomosis OR Ancylostomiasis OR Trichuriosis OR Trichuriasis OR Strongyloidosis OR Strongyloidiasis OR Helminth* OR Ascaris OR Ancylostoma OR Hookworm* OR Trichuris OR Strongyloides OR Alaria OR "rat lungworm" OR "rat lungworms" OR "Echinostoma" OR "Lagochilascaris minor" OR "Zoonotic trypanosomosis" OR Trypanosomiasis OR Chagas OR "Trypanosoma cruzi" OR "Zoonotic intestinal protozoal infection" OR "Zoonotic intestinal protozoal infections" OR Protozoosis OR Protozoasis OR Giardiosis OR Giardiasis OR Cryptosporidiosis OR Blastocystosis OR Sarcocystosis OR Cyclosporiasis OR Cyclospora OR Amoebiasis OR "Amoebic dysentery" OR Entamoeba OR Balantidosis OR Protozoa OR Giardia OR Cryptosporidium OR Blastocystis OR Sarcocystis OR "Cyclospora cayetanensis" OR tiab OR "Entamoeba histolytica" OR "Balantidium coli" OR Trichinellosis OR Trichinosis OR Trichinella OR Toxoplasmosis OR TORCH OR Toxoplasma OR Toxocarosis OR Toxocariasis OR Toxocariosis OR "Larva migrans" OR Toxocara OR Taeniosis OR Taeniasis OR Tapeworm OR Tapeworms OR Taenia OR "Foodborne trematodosis" OR trematodosis OR Trematodiasis OR Fasciolosis OR Fascioliosis OR Fasciolasis OR Fascioliasis OR Distomatosis OR Fasciolopsiasis OR Fasciolopsiosis OR Opisthorchosis OR Opisthorchiasis OR Clonorchiosis OR Clonorchiasis OR Paragonimosis OR Paragonimiasis OR Metagonimus OR Heterophyiasis OR Fluke OR Flukes OR Trematode OR Trematodes OR Fasciola OR Fasciolopsis OR Opisthorchis OR Clonorchis OR Paragonimus OR "Minute intestinal fluke" OR "Minute intestinal flukes" OR "Haplorchis pumilio" OR "Metagonimus yokogawai" OR "Heterophyes" OR Diphyllobothriosis OR Diphyllobothriasis OR Bothriocephalosis OR Bothriocephaliasis OR Diphyllobothrium OR Bothriocephalus OR "Broad tapeworm" OR "Broad tapeworms" OR "Fish tapeworm" OR "Fish tapeworms" OR Cysticercosis OR Neurocysticercosis OR "Taenia solium" OR "Cystic echinococcosis" OR "Hydatid disease" OR "Hydatid diseases" OR Hydatidosis OR "Echinococcus granulosus" OR "Hydatid cyst" OR "Hydatid cysts" OR "Alveolar echinococcosis" OR "Alveolar hydatidosis" OR "Echinococcus multilocularis") AND (fly OR flies OR “filth fly” OR “filth flies” OR Sarcophagidae OR Muscidae OR Calliphoridae OR “household fly” OR “households flies” OR “Musca Domestica” OR “synanthropic fly” OR “synanthropic flies” OR cockroach* OR “Blatella germanica” OR “dung beetle” OR “dung beetles” OR “copraphagic beetle” OR “Copraphagic beetles” OR Onthophagus))
*Limit to scholarly journals</td></tr>
<tr><th>Agriculture Collection</th><th>2/2/21 = 578</th><th>12/27/21 = 0</th></tr>
<tr><td colspan="3">Keyword: "zoonotic parasites” AND"</td></tr>
</table>

<table>
<tr><td colspan="3">Keyword: “fly” OR
Keyword: “cockroach”*OR
Keyword: "dung beetles”
*Limit to peer-reviewed journals</td></tr>
<tr><th>Earth, Atmospheric, and Aquatic Sciences Collection</th><th>2/2/21 = 496</th><th>12/27/21 = 13</th></tr>
<tr><td colspan="3">AB,TI(("Zoonotic enteric pathogen" OR "Zoonotic enteric pathogens" OR "Zoonotic enteric parasite" OR "Zoonotic enteric parasites" OR "Zoonotic parasite" OR "Zoonotic parasites" OR "Animal-to-Human parasite" OR "Animal-to-Human parasites" OR "Foodborne parasite" OR "Foodborne parasites" OR "Waterborne parasite" OR "Waterborne parasites" OR "Water-related parasite" OR "Water-related parasites" OR "Enteric parasite" OR "Enteric parasites" OR parasitism OR "Zoonotic intestinal helminth infection" OR "Zoonotic intestinal helminth infections" OR Helminthosis OR Helminthiasis OR Ascarosis OR Ascariasis OR Ancylostomosis OR Ancylostomiasis OR Trichuriosis OR Trichuriasis OR Strongyloidosis OR Strongyloidiasis OR Helminth* OR Ascaris OR Ancylostoma OR Hookworm* OR Trichuris OR Strongyloides OR Alaria OR "rat lungworm" OR "rat lungworms" OR "Echinostoma" OR "Lagochilascaris minor" OR "Zoonotic trypanosomosis" OR Trypanosomiasis OR Chagas OR "Trypanosoma cruzi" OR "Zoonotic intestinal protozoal infection" OR "Zoonotic intestinal protozoal infections" OR Protozoosis OR Protozoasis OR Giardiosis OR Giardiasis OR Cryptosporidiosis OR Blastocystosis OR Sarcocystosis OR Cyclosporiasis OR Cyclospora OR Amoebiasis OR "Amoebic dysentery" OR Entamoeba OR Balantidosis OR Protozoa OR Giardia OR Cryptosporidium OR Blastocystis OR Sarcocystis OR "Cyclospora cayetanensis" OR tiab OR "Entamoeba histolytica" OR "Balantidium coli" OR Trichinellosis OR Trichinosis OR Trichinella OR Toxoplasmosis OR TORCH OR Toxoplasma OR Toxocarosis OR Toxocariasis OR Toxocariosis OR "Larva migrans" OR Toxocara OR Taeniosis OR Taeniasis OR Tapeworm OR Tapeworms OR Taenia OR "Foodborne trematodosis" OR trematodosis OR Trematodiasis OR Fasciolosis OR Fascioliosis OR Fasciolasis OR Fascioliasis OR Distomatosis OR Fasciolopsiasis OR Fasciolopsiosis OR Opisthorchosis OR Opisthorchiasis OR Clonorchiosis OR Clonorchiasis OR Paragonimosis OR Paragonimiasis OR Metagonimus OR Heterophyiasis OR Fluke OR Flukes OR Trematode OR Trematodes OR Fasciola OR Fasciolopsis OR Opisthorchis OR Clonorchis OR Paragonimus OR "Minute intestinal fluke" OR "Minute intestinal flukes" OR "Haplorchis pumilio" OR "Metagonimus yokogawai" OR "Heterophyes" OR Diphyllobothriosis OR Diphyllobothriasis OR Bothriocephalosis OR Bothriocephaliasis OR Diphyllobothrium OR Bothriocephalus OR "Broad tapeworm" OR "Broad tapeworms" OR "Fish tapeworm" OR "Fish tapeworms" OR Cysticercosis OR Neurocysticercosis OR "Taenia solium" OR "Cystic echinococcosis" OR "Hydatid disease" OR "Hydatid diseases" OR Hydatidosis OR "Echinococcus granulosus" OR "Hydatid cyst" OR "Hydatid cysts" OR "Alveolar echinococcosis" OR "Alveolar hydatidosis" OR "Echinococcus multilocularis") AND (fly OR flies OR “filth fly” OR “filth flies” OR Sarcophagidae OR Muscidae OR Calliphoridae OR “household fly” OR “households flies” OR “Musca Domestica” OR “synanthropic fly” OR “synanthropic flies” OR cockroach* OR “Blatella germanica” OR “dung beetle” OR “dung beetles” OR “copraphagic beetle” OR “Copraphagic beetles” OR Onthophagus))
*Limit to scholarly journals</td></tr>
<tr><th>Environment Complete</th><th>2/2/21 = 467
(not all titles were accessible for upload to citation manager)</th><th>12/27/21 = 19</th></tr>
<tr><td colspan="3">AB(("Zoonotic enteric pathogen" OR "Zoonotic enteric pathogens" OR "Zoonotic enteric parasite" OR "Zoonotic enteric parasites" OR "Zoonotic parasite" OR "Zoonotic parasites" OR "Animal-to-Human parasite" OR "Animal-to-Human parasites" OR "Foodborne parasite" OR "Foodborne parasites" OR "Waterborne parasite" OR "Waterborne parasites" OR "Water-related parasite" OR "Water-related parasites" OR "Enteric parasite" OR "Enteric parasites" OR parasitism OR "Zoonotic intestinal helminth infection" OR "Zoonotic intestinal helminth infections" OR Helminthosis OR Helminthiasis OR Ascarosis OR Ascariasis OR Ancylostomosis OR Ancylostomiasis OR Trichuriosis OR Trichuriasis OR Strongyloidosis OR Strongyloidiasis OR Helminth* OR Ascaris OR Ancylostoma OR Hookworm* OR Trichuris OR Strongyloides OR Alaria OR "rat lungworm" OR "rat lungworms" OR "Echinostoma" OR "Lagochilascaris minor" OR "Zoonotic trypanosomosis" OR Trypanosomiasis OR Chagas OR "Trypanosoma cruzi" OR "Zoonotic intestinal protozoal infection" OR "Zoonotic intestinal protozoal infections" OR Protozoosis OR Protozoasis OR Giardiosis OR Giardiasis OR Cryptosporidiosis OR Blastocystosis OR Sarcocystosis OR Cyclosporiasis OR Cyclospora OR Amoebiasis OR "Amoebic dysentery" OR Entamoeba OR Balantidosis OR Protozoa OR Giardia OR Cryptosporidium OR Blastocystis OR Sarcocystis OR "Cyclospora cayetanensis" OR tiab OR</td></tr>
</table>

<table>
<tr><td colspan="3">"Entamoeba histolytica" OR "Balantidium coli" OR Trichinellosis OR Trichinosis OR Trichinella OR Toxoplasmosis OR TORCH OR Toxoplasma OR Toxocarosis OR Toxocariasis OR Toxocariosis OR "Larva migrans" OR Toxocara OR Taeniosis OR Taeniasis OR Tapeworm OR Tapeworms OR Taenia OR "Foodborne trematodosis" OR trematodosis OR Trematodiasis OR Fasciolosis OR Fascioliosis OR Fasciolasis OR Fascioliasis OR Distomatosis OR Fasciolopsiasis OR Fasciolopsiosis OR Opisthorchosis OR Opisthorchiasis OR Clonorchiosis OR Clonorchiasis OR Paragonimosis OR Paragonimiasis OR Metagonimus OR Heterophyiasis OR Fluke OR Flukes OR Trematode OR Trematodes OR Fasciola OR Fasciolopsis OR Opisthorchis OR Clonorchis OR Paragonimus OR "Minute intestinal fluke" OR "Minute intestinal flukes" OR "Haplorchis pumilio" OR "Metagonimus yokogawai" OR "Heterophyes" OR Diphyllobothriosis OR Diphyllobothriasis OR Bothriocephalosis OR Bothriocephaliasis OR Diphyllobothrium OR Bothriocephalus OR "Broad tapeworm" OR "Broad tapeworms" OR "Fish tapeworm" OR "Fish tapeworms" OR Cysticercosis OR Neurocysticercosis OR "Taenia solium" OR "Cystic echinococcosis" OR "Hydatid disease" OR "Hydatid diseases" OR Hydatidosis OR "Echinococcus granulosus" OR "Hydatid cyst" OR "Hydatid cysts" OR "Alveolar echinococcosis" OR "Alveolar hydatidosis" OR "Echinococcus multilocularis") AND (fly OR flies OR “filth fly” OR “filth flies” OR Sarcophagidae OR Muscidae OR Calliphoridae OR “household fly” OR “households flies” OR “Musca Domestica” OR “synanthropic fly” OR “synanthropic flies” OR cockroach* OR “Blatella germanica” OR “dung beetle” OR “dung beetles” OR “copraphagic beetle” OR “Copraphagic beetles” OR Onthophagus))
*Limit to academic journals</td></tr>
<tr><td>Agricultural and Environmental Science Collection</td><td>2/4/21 = 259</td><td>12/27/21 = 38</td></tr>
<tr><td colspan="3">AB,TI (("Zoonotic enteric pathogen" OR "Zoonotic enteric pathogens" OR "Zoonotic enteric parasite" OR "Zoonotic enteric parasites" OR "Zoonotic parasite" OR "Zoonotic parasites" OR "Animal-to-Human parasite" OR "Animal-to-Human parasites" OR "Foodborne parasite" OR "Foodborne parasites" OR "Waterborne parasite" OR "Waterborne parasites" OR "Water-related parasite" OR "Water-related parasites" OR "Enteric parasite" OR "Enteric parasites" OR parasitism OR "Zoonotic intestinal helminth infection" OR "Zoonotic intestinal helminth infections" OR Helminthosis OR Helminthiasis OR Ascarosis OR Ascariasis OR Ancylostomosis OR Ancylostomiasis OR Trichuriosis OR Trichuriasis OR Strongyloidosis OR Strongyloidiasis OR Helminth* OR Ascaris OR Ancylostoma OR Hookworm* OR Trichuris OR Strongyloides OR Alaria OR "rat lungworm" OR "rat lungworms" OR "Echinostoma" OR "Lagochilascaris minor" OR "Zoonotic trypanosomosis" OR Trypanosomiasis OR Chagas OR "Trypanosoma cruzi" OR "Zoonotic intestinal protozoal infection" OR "Zoonotic intestinal protozoal infections" OR Protozoosis OR Protozoasis OR Giardiosis OR Giardiasis OR Cryptosporidiosis OR Blastocystosis OR Sarcocystosis OR Cyclosporiasis OR Cyclospora OR Amoebiasis OR "Amoebic dysentery" OR Entamoeba OR Balantidosis OR Protozoa OR Giardia OR Cryptosporidium OR Blastocystis OR Sarcocystis OR "Cyclospora cayetanensis" OR tiab OR "Entamoeba histolytica" OR "Balantidium coli" OR Trichinellosis OR Trichinosis OR Trichinella OR Toxoplasmosis OR TORCH OR Toxoplasma OR Toxocarosis OR Toxocariasis OR Toxocariosis OR "Larva migrans" OR Toxocara OR Taeniosis OR Taeniasis OR Tapeworm OR Tapeworms OR Taenia OR "Foodborne trematodosis" OR trematodosis OR Trematodiasis OR Fasciolosis OR Fascioliosis OR Fasciolasis OR Fascioliasis OR Distomatosis OR Fasciolopsiasis OR Fasciolopsiosis OR Opisthorchosis OR Opisthorchiasis OR Clonorchiosis OR Clonorchiasis OR Paragonimosis OR Paragonimiasis OR Metagonimus OR Heterophyiasis OR Fluke OR Flukes OR Trematode OR Trematodes OR Fasciola OR Fasciolopsis OR Opisthorchis OR Clonorchis OR Paragonimus OR "Minute intestinal fluke" OR "Minute intestinal flukes" OR "Haplorchis pumilio" OR "Metagonimus yokogawai" OR "Heterophyes" OR Diphyllobothriosis OR Diphyllobothriasis OR Bothriocephalosis OR Bothriocephaliasis OR Diphyllobothrium OR Bothriocephalus OR "Broad tapeworm" OR "Broad tapeworms" OR "Fish tapeworm" OR "Fish tapeworms" OR Cysticercosis OR Neurocysticercosis OR "Taenia solium" OR "Cystic echinococcosis" OR "Hydatid disease" OR "Hydatid diseases" OR Hydatidosis OR "Echinococcus granulosus" OR "Hydatid cyst" OR "Hydatid cysts" OR "Alveolar echinococcosis" OR "Alveolar hydatidosis" OR "Echinococcus multilocularis") AND (fly OR flies OR “filth fly” OR “filth flies” OR Sarcophagidae OR Muscidae OR Calliphoridae OR “household fly” OR</td></tr>
</table>

<table>
<tr><td colspan="3">“households flies” OR “Musca Domestica” OR “synanthropic fly” OR “synanthropic flies” OR cockroach* OR “Blatella germanica” OR “dung beetle” OR “dung beetles” OR “copraphagic beetle” OR “Copraphagic beetles” OR Onthophagus))
*Limit to scholarly journals</td></tr>
<tr><th>Environmental Studies and Policy</th><th>2/4/21 = 77</th><th>12/27/21 = 59</th></tr>
<tr><td colspan="3">Keyword: "zoonotic parasites” AND"
Keyword: “fly” OR
Keyword: “cockroach”*OR
Keyword: "dung beetles”
*Limit to academic journals</td></tr>
<tr><th>Google Scholar</th><th>2/4/21 =262
(not all titles were accessible for upload to citation manager)</th><th>12/27/21 = 38</th></tr>
<tr><td colspan="3">all abstract ("zoonotic parasite" OR "zoonotic enteric parasite") AND fly OR cockroach OR "dung beetle"</td></tr>
<tr><th>Health and Medicine</th><th>2/4/21 = 1,220</th><th>12/27/21 = 68</th></tr>
<tr><td colspan="3">ab(("Zoonotic enteric pathogen" OR "Zoonotic enteric pathogens" OR "Zoonotic enteric parasite" OR "Zoonotic enteric parasites" OR "Zoonotic parasite" OR "Zoonotic parasites" OR "Animal-to-Human parasite" OR "Animal-to-Human parasites" OR "Foodborne parasite" OR "Foodborne parasites" OR "Waterborne parasite" OR "Waterborne parasites" OR "Water-related parasite" OR "Water-related parasites" OR "Enteric parasite" OR "Enteric parasites" OR parasitism OR "Zoonotic intestinal helminth infection" OR "Zoonotic intestinal helminth infections" OR Helminthosis OR Helminthiasis OR Ascarosis OR Ascariasis OR Ancylostomosis OR Ancylostomiasis OR Trichuriosis OR Trichuriasis OR Strongyloidosis OR Strongyloidiasis OR Helminth* OR Ascaris OR Ancylostoma OR Hookworm* OR Trichuris OR Strongyloides OR Alaria OR "rat lungworm" OR "rat lungworms" OR "Echinostoma" OR "Lagochilascaris minor" OR "Zoonotic trypanosomosis" OR Trypanosomiasis OR Chagas OR "Trypanosoma cruzi" OR "Zoonotic intestinal protozoal infection" OR "Zoonotic intestinal protozoal infections" OR Protozoosis OR Protozoasis OR Giardiosis OR Giardiasis OR Cryptosporidiosis OR Blastocystosis OR Sarcocystosis OR Cyclosporiasis OR Cyclospora OR Amoebiasis OR "Amoebic dysentery" OR Entamoeba OR Balantidosis OR Protozoa OR Giardia OR Cryptosporidium OR Blastocystis OR Sarcocystis OR "Cyclospora cayetanensis" OR tiab OR "Entamoeba histolytica" OR "Balantidium coli" OR Trichinellosis OR Trichinosis OR Trichinella OR Toxoplasmosis OR TORCH OR Toxoplasma OR Toxocarosis OR Toxocariasis OR Toxocariosis OR "Larva migrans" OR Toxocara OR Taeniosis OR Taeniasis OR Tapeworm OR Tapeworms OR Taenia OR "Foodborne trematodosis" OR trematodosis OR Trematodiasis OR Fasciolosis OR Fascioliosis OR Fasciolasis OR Fascioliasis OR Distomatosis OR Fasciolopsiasis OR Fasciolopsiosis OR Opisthorchosis OR Opisthorchiasis OR Clonorchiosis OR Clonorchiasis OR Paragonimosis OR Paragonimiasis OR Metagonimus OR Heterophyiasis OR Fluke OR Flukes OR Trematode OR Trematodes OR Fasciola OR Fasciolopsis OR Opisthorchis OR Clonorchis OR Paragonimus OR "Minute intestinal fluke" OR "Minute intestinal flukes" OR "Haplorchis pumilio" OR "Metagonimus yokogawai" OR "Heterophyes" OR Diphyllobothriosis OR Diphyllobothriasis OR Bothriocephalosis OR Bothriocephaliasis OR Diphyllobothrium OR Bothriocephalus OR "Broad tapeworm" OR "Broad tapeworms" OR "Fish tapeworm" OR "Fish tapeworms" OR Cysticercosis OR Neurocysticercosis OR "Taenia solium" OR "Cystic echinococcosis" OR "Hydatid disease" OR "Hydatid diseases" OR Hydatidosis OR "Echinococcus granulosus" OR "Hydatid cyst" OR "Hydatid cysts" OR "Alveolar echinococcosis" OR "Alveolar hydatidosis" OR "Echinococcus multilocularis") AND (fly OR flies OR “filth fly” OR “filth flies” OR Sarcophagidae OR Muscidae OR Calliphoridae OR “household fly” OR “households flies” OR “Musca Domestica” OR “synanthropic fly” OR “synanthropic flies” OR cockroach* OR “Blatella germanica” OR “dung beetle” OR “dung beetles” OR “copraphagic beetle” OR “Copraphagic beetles” OR Onthophagus))
*Limit to scholarly journals</td></tr>
<tr><th>MEDLINE (Proquest)</th><th>2/4/21 = 1,218</th><th>12/27/21 = 68</th></tr>
<tr><td colspan="3">Ab(("Zoonotic enteric pathogen" OR "Zoonotic enteric pathogens" OR "Zoonotic enteric parasite" OR "Zoonotic enteric parasites" OR "Zoonotic parasite" OR "Zoonotic parasites" OR "Animal-to-Human parasite" OR "Animal-to-Human parasites" OR "Foodborne parasite" OR "Foodborne parasites" OR "Waterborne parasite" OR "Waterborne parasites" OR "Water-related parasite" OR "Water-related parasites" OR "Enteric parasite" OR "Enteric parasites" OR parasitism OR "Zoonotic intestinal helminth infection" OR "Zoonotic</td></tr>
</table>

<table>
<tr><td colspan="3">intestinal helminth infections" OR Helminthosis OR Helminthiasis OR Ascarosis OR Ascariasis OR Ancylostomosis OR Ancylostomiasis OR Trichuriosis OR Trichuriasis OR Strongyloidosis OR Strongyloidiasis OR Helminth* OR Ascaris OR Ancylostoma OR Hookworm* OR Trichuris OR Strongyloides OR Alaria OR "rat lungworm" OR "rat lungworms" OR "Echinostoma" OR "Lagochilascaris minor" OR "Zoonotic trypanosomosis" OR Trypanosomiasis OR Chagas OR "Trypanosoma cruzi" OR "Zoonotic intestinal protozoal infection" OR "Zoonotic intestinal protozoal infections" OR Protozoosis OR Protozoasis OR Giardiosis OR Giardiasis OR Cryptosporidiosis OR Blastocystosis OR Sarcocystosis OR Cyclosporiasis OR Cyclospora OR Amoebiasis OR "Amoebic dysentery" OR Entamoeba OR Balantidosis OR Protozoa OR Giardia OR Cryptosporidium OR Blastocystis OR Sarcocystis OR "Cyclospora cayetanensis" OR tiab OR "Entamoeba histolytica" OR "Balantidium coli" OR Trichinellosis OR Trichinosis OR Trichinella OR Toxoplasmosis OR TORCH OR Toxoplasma OR Toxocarosis OR Toxocariasis OR Toxocariosis OR "Larva migrans" OR Toxocara OR Taeniosis OR Taeniasis OR Tapeworm OR Tapeworms OR Taenia OR "Foodborne trematodosis" OR trematodosis OR Trematodiasis OR Fasciolosis OR Fascioliosis OR Fasciolasis OR Fascioliasis OR Distomatosis OR Fasciolopsiasis OR Fasciolopsiosis OR Opisthorchosis OR Opisthorchiasis OR Clonorchiosis OR Clonorchiasis OR Paragonimosis OR Paragonimiasis OR Metagonimus OR Heterophyiasis OR Fluke OR Flukes OR Trematode OR Trematodes OR Fasciola OR Fasciolopsis OR Opisthorchis OR Clonorchis OR Paragonimus OR "Minute intestinal fluke" OR "Minute intestinal flukes" OR "Haplorchis pumilio" OR "Metagonimus yokogawai" OR "Heterophyes" OR Diphyllobothriosis OR Diphyllobothriasis OR Bothriocephalosis OR Bothriocephaliasis OR Diphyllobothrium OR Bothriocephalus OR "Broad tapeworm" OR "Broad tapeworms" OR "Fish tapeworm" OR "Fish tapeworms" OR Cysticercosis OR Neurocysticercosis OR "Taenia solium" OR "Cystic echinococcosis" OR "Hydatid disease" OR "Hydatid diseases" OR Hydatidosis OR "Echinococcus granulosus" OR "Hydatid cyst" OR "Hydatid cysts" OR "Alveolar echinococcosis" OR "Alveolar hydatidosis" OR "Echinococcus multilocularis") AND (fly OR flies OR “filth fly” OR “filth flies” OR Sarcophagidae OR Muscidae OR Calliphoridae OR “household fly” OR “households flies” OR “Musca Domestica” OR “synanthropic fly” OR “synanthropic flies” OR cockroach* OR “Blatella germanica” OR “dung beetle” OR “dung beetles” OR “copraphagic beetle” OR “Copraphagic beetles” OR Onthophagus))
*Limit to scholarly journals</td></tr>
<tr><th>Nursing and Allied Health Outcomes</th><th>2/4/21 = 292
(not all titles were accessible for upload to citation manager)</th><th>12/27/21 = 0</th></tr>
<tr><td colspan="3"><u>Keyword</u>: "zoonotic parasites” AND"
<u>Keyword</u>: “fly” OR
<u>Keyword</u>: “cockroach”*OR
<u>Keyword</u>: "dung beetles”
*Limit to academic journals</td></tr>
<tr><th>PubMed</th><th>1/28/21 = 1,520</th><th>12/27/21 = 99</th></tr>
<tr><td colspan="3">("Zoonotic enteric pathogen"[tiab] OR "Zoonotic enteric pathogens"[tiab] OR "Zoonotic enteric parasite"[tiab] OR "Zoonotic enteric parasites"[tiab] OR "Zoonotic parasite"[tiab] OR "Zoonotic parasites"[tiab] OR "Animal-to-Human parasite"[tiab] OR "Animal-to-Human parasites"[tiab] OR "Foodborne parasite"[tiab] OR "Foodborne parasites"[tiab] OR "Waterborne parasite"[tiab] OR "Waterborne parasites"[tiab] OR "Water-related parasite"[tiab] OR "Water-related parasites"[tiab] OR "Enteric parasite"[tiab] OR "Enteric parasites"[tiab] OR parasitism[tiab] OR "Zoonotic intestinal helminth infection"[tiab] OR "Zoonotic intestinal helminth infections"[tiab] OR Helminthosis[tiab] OR Helminthiasis[tiab] OR Ascarosis[tiab] OR Ascariasis[tiab] OR Ancylostomosis[tiab] OR Ancylostomiasis[tiab] OR Trichuriosis[tiab] OR Trichuriasis[tiab] OR Strongyloidosis[tiab] OR Strongyloidiasis[tiab] OR Helminth*[tiab] OR Ascaris[tiab] OR Ancylostoma[tiab] OR Hookworm*[tiab] OR Trichuris[tiab] OR Strongyloides[tiab] OR Alaria[tiab] OR "rat lungworm"[tiab] OR "rat lungworms"[tiab] OR "Echinostoma"[tiab] OR "Lagochilascaris minor"[tiab] OR "Zoonotic trypanosomosis"[tiab] OR Trypanosomiasis[tiab] OR Chagas[tiab] OR "Trypanosoma cruzi"[tiab] OR "Zoonotic intestinal protozoal infection"[tiab] OR "Zoonotic intestinal protozoal infections"[tiab] OR Protozoosis[tiab] OR Protozoasis[tiab] OR Giardiosis[tiab] OR Giardiasis[tiab] OR Cryptosporidiosis[tiab] OR Blastocystosis[tiab] OR Sarcocystosis[tiab] OR Cyclosporiasis[tiab] OR Cyclospora[tiab] OR Amoebiasis[tiab] OR "Amoebic dysentery"[tiab] OR Entamoeba[tiab] OR Balantidosis[tiab] OR Protozoa[tiab] OR Giardia[tiab] OR Cryptosporidium[tiab] OR Blastocystis[tiab] OR Sarcocystis[tiab] OR "Cyclospora cayetanensis"[tiab] OR tiab OR "Entamoeba histolytica"[tiab] OR "Balantidium coli"[tiab] OR Trichinellosis[tiab] OR Trichinosis[tiab] OR Trichinella[tiab] OR Toxoplasmosis[tiab] OR TORCH[tiab] OR</td></tr>
</table>

<table>
<tr><td colspan="3">Toxoplasma[tiab] OR Toxocarosis[tiab] OR Toxocariasis[tiab] OR Toxocariosis[tiab] OR "Larva migrans"[tiab] OR Toxocara[tiab] OR Taeniosis[tiab] OR Taeniasis[tiab] OR Tapeworm[tiab] OR Tapeworms[tiab] OR Taenia[tiab] OR "Foodborne trematodosis"[tiab] OR trematodosis[tiab] OR Trematodiasis[tiab] OR Fasciolosis[tiab] OR Fascioliosis[tiab] OR Fasciolasis[tiab] OR Fascioliasis[tiab] OR Distomatosis[tiab] OR Fasciolopsiasis[tiab] OR Fasciolopsiosis[tiab] OR Opisthorchosis[tiab] OR Opisthorchiasis[tiab] OR Clonorchiosis[tiab] OR Clonorchiasis[tiab] OR Paragonimosis[tiab] OR Paragonimiasis[tiab] OR Metagonimus[tiab] OR Heterophyiasis[tiab] OR Fluke[tiab] OR Flukes[tiab] OR Trematode[tiab] OR Trematodes[tiab] OR Fasciola[tiab] OR Fasciolopsis[tiab] OR Opisthorchis[tiab] OR Clonorchis[tiab] OR Paragonimus[tiab] OR "Minute intestinal fluke"[tiab] OR "Minute intestinal flukes"[tiab] OR "Haplorchis pumilio"[tiab] OR "Metagonimus yokogawai"[tiab] OR "Heterophyes"[tiab] OR Diphyllobothriosis[tiab] OR Diphyllobothriasis[tiab] OR Bothriocephalosis[tiab] OR Bothriocephaliasis[tiab] OR Diphyllobothrium[tiab] OR Bothriocephalus[tiab] OR "Broad tapeworm"[tiab] OR "Broad tapeworms"[tiab] OR "Fish tapeworm"[tiab] OR "Fish tapeworms"[tiab] OR Cysticercosis[tiab] OR Neurocysticercosis[tiab] OR "Taenia solium"[tiab] OR "Cystic echinococcosis"[tiab] OR "Hydatid disease"[tiab] OR "Hydatid diseases"[tiab] OR Hydatidosis[tiab] OR "Echinococcus granulosus"[tiab] OR "Hydatid cyst"[tiab] OR "Hydatid cysts"[tiab] OR "Alveolar echinococcosis"[tiab] OR "Alveolar hydatidosis"[tiab] OR "Echinococcus multilocularis"[tiab]) AND
(fly[tiab] OR flies[tiab] OR “filth fly”[tiab] OR “filth flies”[tiab] OR Sarcophagidae[tiab] OR Muscidae[tiab] OR Calliphoridae [tiab] OR “household fly”[tiab] OR “households flies”[tiab] OR “Musca Domestica”[tiab] OR “synanthropic fly”[tiab] OR “synanthropic flies”[tiab] OR cockroach*[tiab] OR “Blatella germanica”[tiab] OR “dung beetle”[tiab] OR “dung beetles”[tiab] OR “copraphagic beetle”[tiab] OR “Copraphagic beetles”[tiab] OR Onthophagus[tiab])</td></tr>
<tr><th>Science Direct</th><th>1/28/21 = 83</th><th>12/27/21 = 7</th></tr>
<tr><td colspan="3">Find articles with these terms:
(“zoonotic parasite” OR “enteric parasite” OR “zoonotic enteric parasite) AND ("fly" OR "cockroach" OR "dung beetle")
*Limit to review and research articles</td></tr>
<tr><th>TOXLINE</th><th>1/28/21 = 27</th><th>12/27/21= 12</th></tr>
<tr><td colspan="3">AB(("Zoonotic enteric pathogen" OR "Zoonotic enteric pathogens" OR "Zoonotic enteric parasite" OR "Zoonotic enteric parasites" OR "Zoonotic parasite" OR "Zoonotic parasites" OR "Animal-to-Human parasite" OR "Animal-to-Human parasites" OR "Foodborne parasite" OR "Foodborne parasites" OR "Waterborne parasite" OR "Waterborne parasites" OR "Water-related parasite" OR "Water-related parasites" OR "Enteric parasite" OR "Enteric parasites" OR parasitism OR "Zoonotic intestinal helminth infection" OR "Zoonotic intestinal helminth infections" OR Helminthosis OR Helminthiasis OR Ascarosis OR Ascariasis OR Ancylostomosis OR Ancylostomiasis OR Trichuriosis OR Trichuriasis OR Strongyloidosis OR Strongyloidiasis OR Helminth* OR Ascaris OR Ancylostoma OR Hookworm* OR Trichuris OR Strongyloides OR Alaria OR "rat lungworm" OR "rat lungworms" OR "Echinostoma" OR "Lagochilascaris minor" OR "Zoonotic trypanosomosis" OR Trypanosomiasis OR Chagas OR "Trypanosoma cruzi" OR "Zoonotic intestinal protozoal infection" OR "Zoonotic intestinal protozoal infections" OR Protozoosis OR Protozoasis OR Giardiosis OR Giardiasis OR Cryptosporidiosis OR Blastocystosis OR Sarcocystosis OR Cyclosporiasis OR Cyclospora OR Amoebiasis OR "Amoebic dysentery" OR Entamoeba OR Balantidosis OR Protozoa OR Giardia OR Cryptosporidium OR Blastocystis OR Sarcocystis OR "Cyclospora cayetanensis" OR tiab OR "Entamoeba histolytica" OR "Balantidium coli" OR Trichinellosis OR Trichinosis OR Trichinella OR Toxoplasmosis OR TORCH OR Toxoplasma OR Toxocarosis OR Toxocariasis OR Toxocariosis OR "Larva migrans" OR Toxocara OR Taeniosis OR Taeniasis OR Tapeworm OR Tapeworms OR Taenia OR "Foodborne trematodosis" OR trematodosis OR Trematodiasis OR Fasciolosis OR Fascioliosis OR Fasciolasis OR Fascioliasis OR Distomatosis OR Fasciolopsiasis OR Fasciolopsiosis OR Opisthorchosis OR Opisthorchiasis OR Clonorchiosis OR Clonorchiasis OR Paragonimosis OR Paragonimiasis OR Metagonimus OR Heterophyiasis OR Fluke OR Flukes OR Trematode OR Trematodes OR Fasciola OR Fasciolopsis OR Opisthorchis OR Clonorchis OR Paragonimus OR "Minute intestinal fluke" OR "Minute intestinal flukes" OR "Haplorchis pumilio" OR "Metagonimus yokogawai" OR "Heterophyes" OR Diphyllobothriosis OR Diphyllobothriasis OR Bothriocephalosis OR Bothriocephaliasis OR Diphyllobothrium OR Bothriocephalus OR "Broad tapeworm" OR "Broad tapeworms" OR "Fish tapeworm" OR "Fish tapeworms" OR Cysticercosis OR Neurocysticercosis OR "Taenia solium" OR "Cystic echinococcosis" OR "Hydatid disease" OR "Hydatid diseases" OR Hydatidosis OR "Echinococcus granulosus" OR "Hydatid cyst" OR "Hydatid cysts" OR "Alveolar echinococcosis" OR "Alveolar hydatidosis" OR "Echinococcus</td></tr>
</table>

<table>
<tr><td colspan="3">multilocularis") AND (fly OR flies OR “filth fly” OR “filth flies” OR Sarcophagidae OR Muscidae OR Calliphoridae OR “household fly” OR “households flies” OR “Musca Domestica” OR “synanthropic fly” OR “synanthropic flies” OR cockroach* OR “Blatella germanica” OR “dung beetle” OR “dung beetles” OR “copraphagic beetle” OR “Copraphagic beetles” OR Onthophagus))
*Limit to scholarly journals</td></tr>
<tr><th>Web of Science Core Collection</th><th>1/28/21 = 2,207</th><th>12/27/21 = 136</th></tr>
<tr><td colspan="3">AB= (("Zoonotic enteric pathogen" OR "Zoonotic enteric pathogens" OR "Zoonotic enteric parasite" OR "Zoonotic enteric parasites" OR "Zoonotic parasite" OR "Zoonotic parasites" OR "Animal-to-Human parasite" OR "Animal-to-Human parasites" OR "Foodborne parasite" OR "Foodborne parasites" OR "Waterborne parasite" OR "Waterborne parasites" OR "Water-related parasite" OR "Water-related parasites" OR "Enteric parasite" OR "Enteric parasites" OR parasitism OR "Zoonotic intestinal helminth infection" OR "Zoonotic intestinal helminth infections" OR Helminthosis OR Helminthiasis OR Ascarosis OR Ascariasis OR Ancylostomosis OR Ancylostomiasis OR Trichuriosis OR Trichuriasis OR Strongyloidosis OR Strongyloidiasis OR Helminth* OR Ascaris OR Ancylostoma OR Hookworm* OR Trichuris OR Strongyloides OR Alaria OR "rat lungworm" OR "rat lungworms" OR "Echinostoma" OR "Lagochilascaris minor" OR "Zoonotic trypanosomosis" OR Trypanosomiasis OR Chagas OR "Trypanosoma cruzi" OR "Zoonotic intestinal protozoal infection" OR "Zoonotic intestinal protozoal infections" OR Protozoosis OR Protozoasis OR Giardiosis OR Giardiasis OR Cryptosporidiosis OR Blastocystosis OR Sarcocystosis OR Cyclosporiasis OR Cyclospora OR Amoebiasis OR "Amoebic dysentery" OR Entamoeba OR Balantidosis OR Protozoa OR Giardia OR Cryptosporidium OR Blastocystis OR Sarcocystis OR "Cyclospora cayetanensis" OR tiab OR "Entamoeba histolytica" OR "Balantidium coli" OR Trichinellosis OR Trichinosis OR Trichinella OR Toxoplasmosis OR TORCH OR Toxoplasma OR Toxocarosis OR Toxocariasis OR Toxocariosis OR "Larva migrans" OR Toxocara OR Taeniosis OR Taeniasis OR Tapeworm OR Tapeworms OR Taenia OR "Foodborne trematodosis" OR trematodosis OR Trematodiasis OR Fasciolosis OR Fascioliosis OR Fasciolasis OR Fascioliasis OR Distomatosis OR Fasciolopsiasis OR Fasciolopsiosis OR Opisthorchosis OR Opisthorchiasis OR Clonorchiosis OR Clonorchiasis OR Paragonimosis OR Paragonimiasis OR Metagonimus OR Heterophyiasis OR Fluke OR Flukes OR Trematode OR Trematodes OR Fasciola OR Fasciolopsis OR Opisthorchis OR Clonorchis OR Paragonimus OR "Minute intestinal fluke" OR "Minute intestinal flukes" OR "Haplorchis pumilio" OR "Metagonimus yokogawai" OR "Heterophyes" OR Diphyllobothriosis OR Diphyllobothriasis OR Bothriocephalosis OR Bothriocephaliasis OR Diphyllobothrium OR Bothriocephalus OR "Broad tapeworm" OR "Broad tapeworms" OR "Fish tapeworm" OR "Fish tapeworms" OR Cysticercosis OR Neurocysticercosis OR "Taenia solium" OR "Cystic echinococcosis" OR "Hydatid disease" OR "Hydatid diseases" OR Hydatidosis OR "Echinococcus granulosus" OR "Hydatid cyst" OR "Hydatid cysts" OR "Alveolar echinococcosis" OR "Alveolar hydatidosis" OR "Echinococcus multilocularis") AND (fly OR flies OR “filth fly” OR “filth flies” OR Sarcophagidae OR Muscidae OR Calliphoridae OR “household fly” OR “households flies” OR “Musca Domestica” OR “synanthropic fly” OR “synanthropic flies” OR cockroach* OR “Blatella germanica” OR “dung beetle” OR “dung beetles” OR “copraphagic beetle” OR “Copraphagic beetles” OR Onthophagus))
*Limited to article, review, or early access</td></tr>
<tr><td>Total</td><td>9,577</td><td>579</td></tr>
<tr><td>Accessible Total</td><td>9,484</td><td>579</td></tr>
</table>

Full number of titles screened= 10,063
